# Linker Chemistry in Radiopharmaceutical Design

**DOI:** 10.1021/acs.bioconjchem.6c00056

**Published:** 2026-03-26

**Authors:** Ava Stoddard, Kiarra Furey, Gina Dehlavi, Mayuresh M. Mane, Joni Sebastiano, Brian M. Zeglis

**Affiliations:** † Department of Chemistry, Hunter College, 5924 City University of New York, New York, New York 10065, United States; ‡ Ph.D. Program in Biology, Graduate Center of City University of New York, New York, New York 10016, United States; § Department of Radiology, Memorial Sloan Kettering Cancer Center, New York, New York 10065, United States; ∥ Ph.D. Program in Biochemistry, Graduate Center of City University of New York, New York, New York 10016, United States; ⊥ Department of Radiology, Weill Cornell Medical College, New York, New York 10065, United States

## Abstract

Over the last half century, molecularly targeted bioconjugates have revolutionized nuclear medicine. An array of vectors, ranging from small molecule peptidomimetics to macromolecular immunoglobulins, has been radiolabeled with radionuclides to create probes that have been deployed in both the laboratory and the clinic for positron emission tomography (PET), single photon emission computed tomography (SPECT), and radiopharmaceutical therapy. Generally speaking, these compounds have four constituent parts: (i) a targeting vector; (ii) a radionuclide; (iii) a labeling moiety, such as a chelator for radiometals or a prosthetic group for radiohalogens; and (iv) a linker that connects the vector and the labeling moiety. Each of these parts is critical to the performance of the radiopharmaceutical, but the importance of the former—the linker—can be lost in the shadow of its flashier teammates. Strictly speaking, the linker’s job is simple: stably connect the vector and the radiolabeling moiety so that they do not become detached *in vivo*. However, recent years have been a witness to increasing efforts to exploit the properties of linkers to improve the pharmacokinetic profiles of radiopharmaceuticals. Along these lines, the most common strategies are predicated on changing the structure of the linker to alter the hydrophobicity and bioavailability of the probe, but several other innovative approaches have emerged as well, including those that rely upon stimuli for the *cleavage* of the linker. In this review, we will offer a systematic and critical discussion of the ways in which radiopharmaceutical chemists have leveraged linker chemistry to optimize the *in vivo* performance of probes for nuclear imaging and therapy, with a particular emphasis on nascent methodologies. We will also explore the lessons that other fields, most notably the development of antibody-drug conjugates, can offer the radiopharmaceutical community with respect to the design and implementation of new linker technologies.

## Graphical abstract

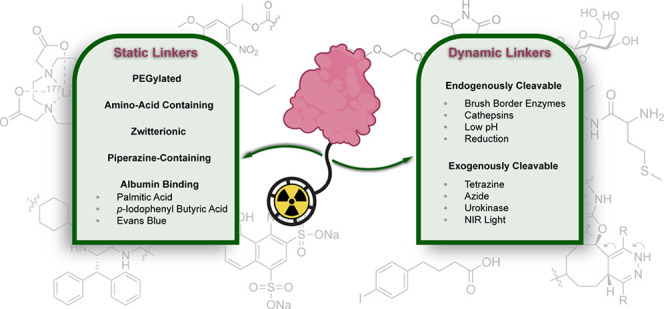

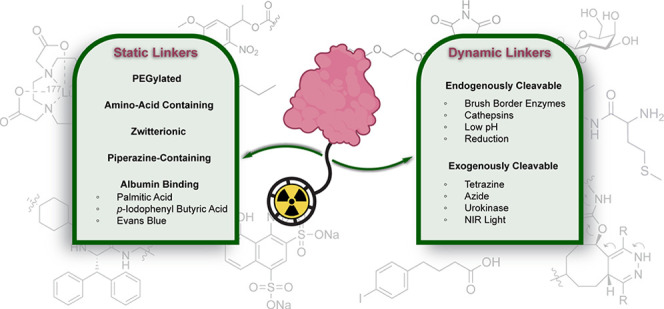

## Introduction

Over the last three decades, molecularly targeted radiopharmaceuticals have transformed nuclear medicine. Vectors ranging from small molecules to nanoparticles have been labeled with radionuclides spanning carbon-11 to thorium-227 for positron emission tomography (PET), single photon emission computed tomography (SPECT), and radiopharmaceutical therapy for an array of diseases. While nuclear medicine plays critical roles in cardiology, neurology, and infectious disease, its greatest utility and potential lie in oncology. In this context, the recent advent of the concept of theranostics has further cemented the position of nuclear imaging and radiopharmaceutical therapy in the clinical mainstream, a shift reinforced by the recent FDA approval of probes for the imaging and treatment of neuroendocrine tumors (i.e., [^68^Ga]­Ga-DOTA-TATE and [^177^Lu]­Lu-DOTA-TATE) and prostate cancer (i.e., [^18^F]­DCFPyL, [^68^Ga]­Ga-PSMA-11, and [^177^Lu]­Lu-PSMA-617).

Generally speaking, modern radiopharmaceuticals take one of two forms: directly radiolabeled probes and radioconjugates. In the former, the radionuclide itself—and *only* the radionuclide—is covalently attached to the targeting vector, typically a small molecule but occasionally a peptide or protein. This approach facilitates the creation of agents that are structurally similar to (or even isotopologues of) the parent vector; however, its use is limited to radiohalogens (e.g., fluorine-18, iodine-131) as well as radioisotopes of carbon (e.g., carbon-11) and nitrogen (e.g., nitrogen-13). Examples of this class of radiopharmaceuticals include [^18^F]­fluoroestradiol, [^131^I]­metaiodobenzylguanidine, ^11^C-labeled Pittsburgh Compound B, [^13^N]­glutamate, and several immunoglobulins labeled with iodine-124 or iodine-131 via the direct radioiodination of tyrosine residues (e.g., [^131^I]­I-3F8).

The latter class of radiopharmaceuticals—radioconjugates—will be our focus here. The anatomy of a radioconjugate can be distilled into four constituent parts: (i) a targeting vector; (ii) a radionuclide; (iii) a labeling moiety, such as a chelator or prosthetic group; and (iv) a linker that connects the vector and the labeling moiety (Figure 1). Each of these components is vital to the *in vivo* behavior of the radiopharmaceutical, yet the value of the linker can be lost in the shadow of its flashier compatriots. Indeed, the first three (i.e., the vector, the radionuclide, and the chelator or prosthetic group) are often the principal sources of concern during the development of a novel radiopharmaceutical, and each has been reviewed extensively.^1^ In contrast, the linker can often be an afterthought. On some level, this is understandable. The linker’s job is not glamorous. It does not bind the target (i.e., the vector), it does not emit radiation (i.e., the nuclide), and it is not directly responsible for the star of the show (i.e., the chelator or prosthetic group). Instead, the linker’s primary job is as critical as it is mundane: to stably connect the vector and the radiolabel so that they do not become detached *in vivo*. That said, in recent years, radiopharmaceutical chemists have increasingly looked to exploit linkers in ways that look beyond their role as simple molecular tethers. Along these lines, a significant—and increasing—amount of research has been dedicated to leveraging linkers to improve the pharmacokinetic profiles of radiopharmaceuticals, specifically increasing their target uptake and decreasing their background accretion and retention. The most common strategies are predicated on changing the structure of the linker to alter the hydrophobicity and bioavailability of the probe, but several other innovative approaches have emerged as well, including those that rely upon external or internal stimuli to facilitate the temporally and spatially resolved *cleavage* of the linker.

**Figure 1:**
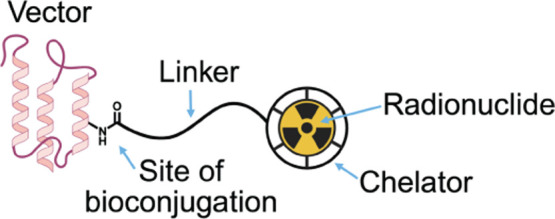
Anatomy of a radiolabeled bioconjugate.

Despite the rapid growth of interest in this area, there are no extant reviews that cover the role of linkers in radiopharmaceutical chemistry and nuclear medicine. As an antidote, we seek herein to offer a systematic and critical discussion of the ways in which linker chemistry has been harnessed to improve the performance of radiopharmaceuticals. The review will be divided into two sections. We will first address *static* linkers, those meant to remain intact throughout the radiopharmaceutical’s transit in the body. Then, we progress to a discussion of *dynamic* linkers that are meant to release their cargo *in vivo*. Of course, the review will not—and, realistically, cannot—be comprehensive, but representative examples of each type of linker will be discussed. Particular emphasis will be placed on comparative studies and nascent methodologies. When appropriate, we will also explore the lessons and inspiration that other fields, most notably the development of antibody-drug conjugates, can offer the radiopharmaceutical community with respect to the design and implementation of new linker technologies. Finally, it is important to note that we will *not* cover the stochastic, site-selective, and site-specific bioconjugation strategies that are used to attach these linkers to vectors, chelators, and prosthetic groups, as this topic has been thoroughly and ably covered elsewhere.^2–4^

## Static Linkers

### PEGylated Linkers

The most straightforward structure for a linker between a vector and a chelator or prosthetic group is obviously a linear chain of atoms. This is accomplished most simply via an aliphatic carbon chain [i.e., −(CH_2_)_*n*_−], but such structures can increase the hydrophobicity of probes and impair their *in vivo* behavior.^5^ As a result, radiopharmaceutical chemists (and pharmaceutical scientists more broadly) have frequently turned to linkers containing poly­(ethylene glycol) (PEG) motifs as more hydrophilic alternatives. Indeed, over the years, the use of linkers containing multiple ethylene glycol units has repeatedly been shown to bolster the pharmacokinetic and pharmacodynamic profiles of both ADCs and radiopharmaceuticals by improving solubility, decreasing immunogenicity, and protecting payloads.^6^

To begin with the fundamentals, the term “polyethylene glycol” describes a polymer of ethylene oxide monomers. PEG chains can vary in size (from ∼100 Da up to ∼400 000 Da), can be linear or branched, and can be mono- or polydisperse. However, in the context of (radio)­pharmaceutical linkers, monodisperse, linear chains of between 2 and 20 units are most common. In truth, it would probably be more appropriate to call these short chains “*oligo*ethylene glycol linkers”, but—except for a handful of instances of particularly rigorous nomenclature^7^—the field has collectively decided to use the colloquialism “PEG linkers” nonetheless.

The biomedical use of PEG linkers was pioneered in the 1970s by Robert B. Davis at Rutgers University, who found that the stochastic attachment of PEG linkers to bovine liver catalase dramatically increased its circulation time compared to unmodified catalase.^8^ The earliest applications of PEG chains to radiopharmaceutical chemistry can be traced back to the mid-to-late 1990s, when a number of laboratories experimented with the PEGylation of radiolabeled antibodies, liposomes, and nanoparticles to improve their pharmacokinetic profiles.^9,10^ The advent of PEG-based *linkers* followed a few years later in the early aughts. In 2001, for example, Wen et al. created an EGF-targeting radioimmunoconjugate in which a PEG linker bridged the antibody (i.e., C225) and an [^111^In]­In-DTPA group and found that this radioimmunoconjugate afforded better tumor-to-liver activity concentration ratios than an analogous agent without a PEG linker.^8^ Soon thereafter, Chen, Conti, and coworkers incorporated PEG linkers into^18^F- and ^64^Cu-labeled variants of c­(RGDyK) for the PET imaging of α_v_β_3_ expression in tumor tissue and found that the PEG-bearing probes exhibited improved tumor retention and pharmacokinetic profiles compared to analogues without PEG linkers.^11,12^

In the cases described above, the PEG linkers were quite large (i.e., molecular weight = 3,400 DA; ∼77 ethylene oxide units). While the manuscripts do not explicitly comment upon the dispersity of these polymers, it is relatively unlikely that they are monodisperse. In the two decades since these earliest investigations, the field has increasingly turned to shorter, monodisperse PEG linkers composed of 1–20 ethyl oxide units. Examples abound in which these linkers have been leveraged to alter the *in vivo* performance of radiopharmaceuticals. However, it is important to note that while PEG linkers *often* improve the pharmacokinetic profiles of radiopharmaceuticals, this is not *always* the case. PEG linkers of different lengths can exert different influences on the same radiopharmaceutical framework, and sometimes, the addition of a PEG linker can even be deleterious.^13–17^ Furthermore, in some cases, PEG linkers can be immunogenic and lead to the production of anti-PEG antibodies.^18^

In 2014, Jamous and coworkers performed a beautifully systematic study on the influence of PEG linkers of various lengths on ^177^Lu-labeled variants of a linear gastrin-releasing peptide receptor-targeting peptide.^19^ More specifically, the team synthesized probes with PEG_2_, PEG_4_, PEG_6_, and PEG_12_ linkers and found that *in vitro* stability increased dramatically from PEG_2_ (*t*_1/2_ in serum = ∼250 h) to PEG_4_ (*t*_1/2_ = ∼410 h) to PEG_6_ (*t*_1/2_ = ∼580 h) without sacrificing binding affinity (IC_50_ values of 8.6 ± 2.7, 6.2 ± 0.6, and 9.9 ± 2.9 nM, respectively). Interestingly, however, extending the linker to 12 units sacrificed some stability (*t*_1/2_ = ∼410 h) and binding affinity (IC_50_ = 15.0 ± 5.3 nM) compared to those of the PEG_6_-bearing variant. Subsequent acute biodistribution studies in a murine model of GRPR-positive prostate cancer revealed that the [^177^Lu]­Lu-DO3A-PEG_4_- and [^177^Lu]­Lu-DO3A-PEG_6_-labeled probes exhibited excellent *in vivo* performance with high tumor uptake and retention and comparable tumor-to-healthy organ activity concentration ratios.

A 2019 study by Rondon et al. underscores the degree to which PEG linkers can alter the elimination rate and excretion pathway of radiotracers.^20^ As part of their efforts to develop a new strategy for the pretargeted radioimmunotherapy of peritoneal carcinomatosis, the team synthesized three ^177^Lu-labeled DOTA-dipyridyltetrazines with PEG_3_, PEG_7_, and PEG_11_ linkers. Upon intraperitoneal injection into healthy mice, the shift from the PEG_3_ linker to the PEG_7_ and PEG_11_ variants was observed to dramatically accelerate the clearance of the radioligands and shunt their excretion from the renal *and* hepatobiliary systems (i.e., liver, large intestine, and small intestine) to the kidneys alone. Subsequent longitudinal therapy studies in mice with peritoneal carcinomatosis revealed that pretargeting with a *trans*-cyclooctene (TCO)-bearing CEA-targeting mAb and the [^177^Lu]­Lu-DOTA-PEG_7_-labeled dipyridyltetrazine effectively reduced the peritoneal carcinomatosis index (PCI) compared to the administration of saline or the radioligand alone.

More recently, Lee et al. interrogated the effects of inserting PEG_2_ and PEG_4_ linkers into Tyr^3^‑octreotide-based probes bearing a lead-specific chelator (PSC) for radiolabeling with lead-203 (for PET) and lead-212 (for α-particle therapy) (Figure 2).^21^ A peptide conjugate bearing a PEG_2_ linker (i.e., PSC-PEG_2_-TOC) exhibited slightly increased affinity (IC_50_ = 5.3 ± 1.2 nM) compared to the parental conjugate (i.e., PSC-TOC; 6.2 ± 1.1 nM), while the variant with a PEG_4_ linker (i.e., PSC-PEG_4_-TOC) displayed a significant drop in affinity (9.4 ± 1.3 nM). Subsequent *in vitro* and *in vivo* experiments revealed that [^203^Pb]­Pb-PSC-PEG_2_-TOC provides a higher rate of target-specific cellular uptake than [^203^Pb]­Pb-PSC-TOC, as well as dramatically enhanced tumor-to-kidney activity concentration ratios in mice bearing subcutaneous AR42J xenografts, and [^212^Pb]­Pb-PSC-PEG_2_-TOC exhibited a dose-dependent therapeutic response in the same murine model.

**Figure 2:**
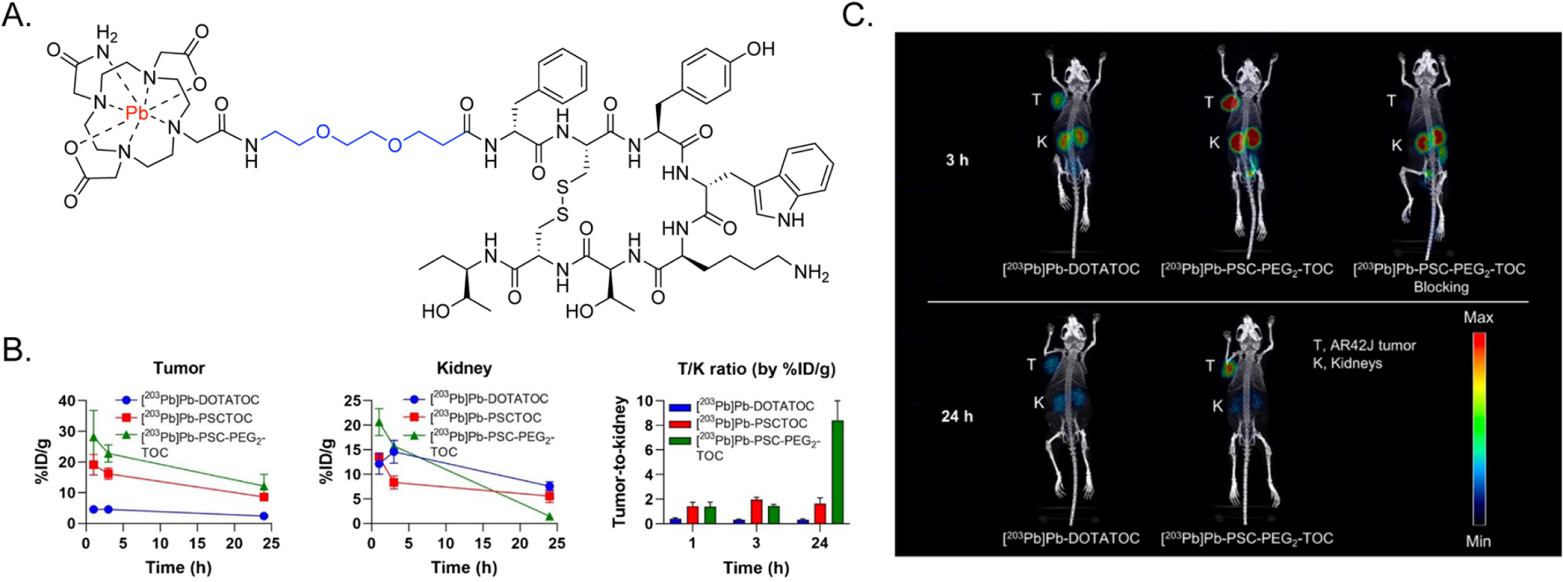
(A) Chemical structure of [^203/212^Pb]­Pb-PSC-PEG_2_-TOC, with the radiometal in red and the PEG linker in blue. (B) Biodistribution data acquired with [^203^Pb]­Pb-DOTATOC, [^203^Pb]­Pb-PSCTOC, and [^203^Pb]­Pb-PSC-PEG_2_-TOC in athymic nude mice bearing AR42J xenografts. (C) SPECT/CT images of athymic nude mice bearing AR42J xenografts acquired 3 and 24 h after the intravenous administration of [^203^Pb]­Pb-DOTATOC and [^203^Pb]­Pb-PSC-PEG_2_-TOC. Adapted and reprinted from ref^21^ under a Creative Commons License (CC BY 4.0).

In 2024, Bobba et al. developed a trio of CD46-targeting radioimmunoconjugates in which PEG_0_, PEG_4_, and PEG_8_ linkers bridged the antibody (i.e., YS5) and a macropa chelator for imaging and therapy with the emergent theranostic pair cesium-134/actinium-225.^22^ In a murine model of CD46-positive prostate cancer, [^225^Ac]­Ac-macropa-PEG_4_-YS5 produced tumoral activity concentrations dramatically higher than those of the variants with PEG_0_ and PEG_8_ linkers, as well as improved tumor-to-muscle and tumor-to-kidney activity concentration ratios, and longitudinal therapy studies in the same mouse model revealed that treatment with [^225^Ac]­Ac-macropa-PEG_4_-YS5 yielded longer median survival times than treatment with [^225^Ac]­Ac-DOTA-YS5. Curiously, however, similar differences in tumoral uptake and tumor-to-background contrast were *not* seen in PET images acquired with ^134^Ce-labeled PEG_0_-, PEG_4_-, and PEG_8_-YS5.

Finally, before we move on, it is important to note that a small but growing body of literature suggests that PEG linkers can have a role in enhancing the metabolism of a radiopharmaceutical and reducing its nonspecific uptake. For example, Guillou et al. elegantly illustrated that a radioimmunoconjugate bearing a short PEG linker (i.e., [^89^Zr]­Zr-DFO-PEG_3_-azepin-mAb) not only had improved aqueous phase solubility and radiochemical conversion than an analogous probe without a PEG linker (i.e., [^89^Zr]­Zr-DFO-azepin-mAb) but also exhibited improved target-to-background contrast.^23^ The team posited that the PEG linker accelerates the excretion of the entire construct, largely because the degradation of the PEG_3_ chain produces metabolites that are rapidly cleared from the body.

### Alternatives to PEG Linkers

While PEG linkers have proven effective at modulating the pharmacokinetic and pharmacodynamic profiles of radiopharmaceuticals, several other classes of static linkers have emerged as alternatives that offer enhanced rigidity, hydrophilicity, and modularity.

#### Piperazine-Containing Linkers

Piperazine (PIP) is a 6-atom heterocycle that introduces rigidity, basicity, and moderate lipophilicity into linker architectures. The p*K*_a_ values of its two endocyclic nitrogens are ∼9 and ∼5 (depending on substitution patterns), so at least one will typically be protonated at physiological pH, giving piperazine-containing linkers a positive charge. Furthermore, these two contraposed nitrogens offer convenient handles for functionalization.^24,25^ Several well-known radiopharmaceuticals contain piperazine moieties—including a handful of radiolabeled FAP inhibitors (including [^68^Ga]­Ga-FAPI-04 and [^68^Ga]­Ga-FAPI-46)—but systematic studies unraveling the impact of piperazines *within linkers* remain relatively rare (Figure 3).^26–28^ That said, Aso et al. performed a comparative study in 2023 exploring the biodistribution of ^131^I- and ^211^At-labeled FAP inhibitors bearing PEG and piperazine linkers and found that the probes containing the two linkers performed relatively similarly in murine models of FAP-expressing lung cancer.^29^ A few years earlier, Mansour et al. interrogated the *in vitro* and *in vivo* properties of ^64^Cu-labeled GRPR-targeting peptides with PEG-, piperazine-, and hexanedioic acid-containing linkers.^30^ The team found that the variant with the dianionic linker (i.e., [^64^Cu]­Cu-NOTA-AHDA-RM26) boasted a significantly reduced binding affinity for GRPR (18.0 ± 2.8 nM) compared to the PEG- and piperazine-bearing probes (1.0 ± 0.5 and 1.0 ± 2.0 nM, respectively). However, all three imaging agents proved to be stable *in vivo* and boasted relatively similar pharmacokinetic profiles in mice bearing GRPR-expressing PC-3 prostate cancer xenografts, suggesting that (at least in this case) the linker does not play a dramatic role in determining *in vivo* performance.

**Figure 3:**
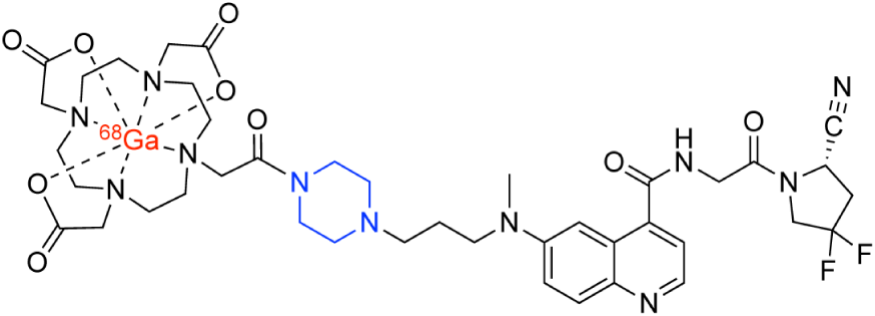
Structure of [^68^Ga]­Ga-FAPI-46 with a piperazine linker in blue.

#### Zwitterionic Linkers

Zwitterions are neutral molecules that contain an identical number of isolated positively and negatively charged moieties. This array of charges gives zwitterionic polymers a strong affinity for water molecules, significantly enhancing their hydrophilicity (and the hydrophilicity of molecules to which they are attached). The potential of zwitterionic linkers as tools for pharmacokinetic optimization has not gone unnoticed. To wit, Maynard et al. conjugated a zwitterionic polymer called poly­(caprolactone-carboxybetaine) (pCLZ) to a human growth hormone receptor antagonist (GHA); attached fluorine-18 to bare GHA, bare pCLZ, and the GHA-pCLZ conjugate; and then used PET imaging to demonstrate that the zwitterionic polymer lengthens the circulation time of the conjugate (*t*_1/2_ = 0.74 h) compared to the protein alone (*t*_1/2_ = 0.36 h).^31^

In the case described above, the zwitterion is (of course) *not* used as a linker. And indeed, examples of radiopharmaceuticals with zwitterionic linkers are—at least currently—few and far between, underscoring the emergent nature of this technology. In 2025, Yuan et al. reported [^68^Ga]­Ga-FAPI-BN-1, a^68^Ga-labeled fibroblast activation protein (FAP)-targeted probe with a linker containing a boron trifluoride zwitterion (Figure 4).^32^ Solubility assays revealed that [^68^Ga]­Ga-FAPI-BN-1 was more hydrophilic (logP = −3.3 ± 0.1) than a close cousin lacking the zwitterionic linker ([^68^Ga]­Ga-FAPI-N; logP = −3.1 ± 0.1) as well as the ubiquitous [^68^Ga]­Ga-FAPI-04 (logP = −3.0 ± 0.1) and was taken up more rapidly in FAP-expressing U87MG cells. Subsequent PET imaging experiments in mice bearing subcutaneous U87MG xenografts revealed that [^68^Ga]­Ga-FAPI-BN-1 dramatically outperformed both [^68^Ga]­Ga-FAPI-N *and* [^68^Ga]­Ga-FAPI-04, yielding greater tumoral accretion and better tumor-to-healthy tissue activity concentration ratios than both. This can be seen when comparing [^68^Ga]­Ga-FAPI-BN-1 uptake in the tumor 120 min postinjection (23.5 ± 2.9 %ID/g) to that of [^68^Ga]­Ga-FAPI-N and [^68^Ga]­Ga-FAPI-04 at the same time point (12.7 ± 1.8 %ID/g and 7.6 ± 1.1 %ID/g, respectively). Finally, the Schnermann laboratory at the National Institutes of Health developed a zwitterionic benzyl α-ammonium carbonate-based linker as a more hydrophilic alternative to the self-immolative *para*-aminobenzyl (PAB) linker employed in many cleavable ADCs (*vide infra*), though this architecture has yet to be used in radioconjugates.^33^

**Figure 4:**
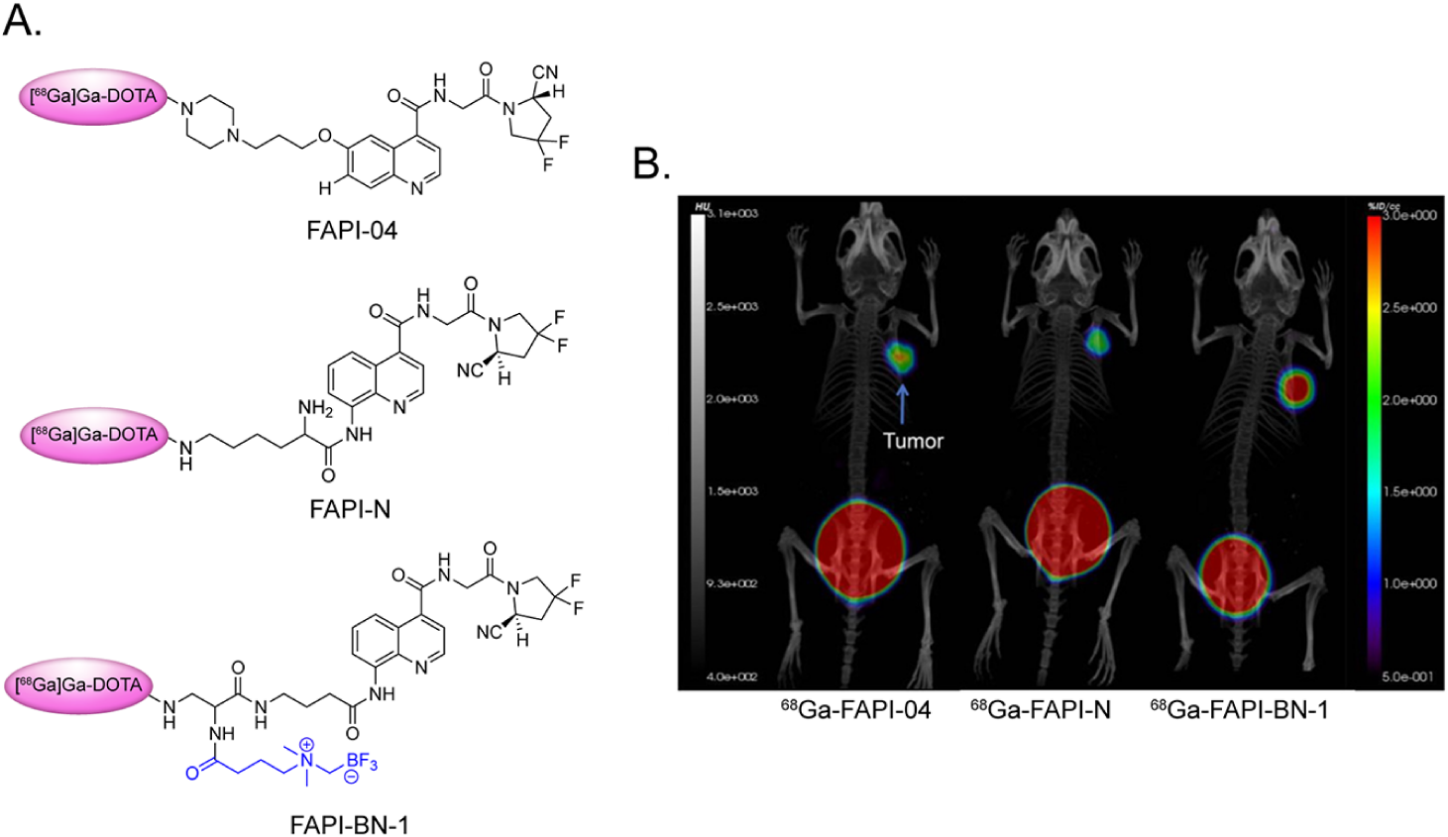
(A) Structures of [^68^Ga]­Ga-DOTA-FAPI-04, [^68^Ga]­Ga-DOTA-FAPI-N, and [^68^Ga]­Ga-DOTA-FAPI-BN-1 with the zwitterionic linker of the latter in blue. (B) Coronal PET/CT images acquired 1 h after the administration of the three radiotracers to athymic nude mice bearing U87MG tumors. Adapted and reprinted with permission from ref^32^.

#### Amino Acid-Containing Linkers

Linkers based on short chains of amino acids have also attracted attention in the field due to the wide variety of physicochemical characteristics offered by the various side chains (e.g., hydrophobicity, charge, etc.), as well as the ease of solid phase synthesis. Almost 20 years ago, for example, Parry et al. interrogated the *in vitro* and *in vivo* properties of ^64^Cu-DOTA-labeled variants of bombesin containing five different tripeptide linkers: GGG, GSG, GSS, GEG, and GEE.^34^ The negative charge introduced by the GEG and GEE linkers reduced the IC_50_ values of the peptides by 1 and 2 orders of magnitude, respectively, precluding further experimentation. However, PET imaging and biodistribution studies with the remaining probes in a murine model of prostate cancer revealed that the trio exhibited similar pharmacokinetic profiles and uptake patterns. More dramatic differences in *in vivo* performance were highlighted in a more recent structure–activity relationship study by Meyer et al.^35^ This work centered on the synthesis of a library of ^68^Ga- and ^18^F-labeled tetrazine radioligands for *in vivo* pretargeting, including variants with linkers composed of sequential lysine residues and others with linkers containing PEG_7_ chains in addition to single amino acids (i.e., lysine, histidine, arginine, and aspartic acid). *In vitro* and *in vivo* analyses revealed that both types of amino acid-containing linkers dramatically decreased the LogD values and blood residence times of the radioligands compared to analogues bearing PEG-only linkers, physicochemical properties that ultimately made the former less suitable for *in vivo* pretargeting.

PSMA-targeting probes have provided especially fertile ground for amino acid-containing linkers.^36^ Just last year, Liu et al. created a library of ^68^Ga-labeled PSMA-targeting probes with linkers containing β-branched aromatic α-amino acids within the linkers.^37^ These probes exhibited binding affinities for PSMA comparable to that of [^68^Ga]­Ga-PSMA-617 but dramatically different pharmacokinetic profiles, including reduced retention in the kidneys and (in one case) elevated uptake in tumor tissue. Finally, Liolios et al. developed a family of bispecific PSMA- and GRPR-targeted ^68^Ga-labeled radiotracers with linkers containing 1, 2, or 3 HE dipeptide units containing both positive (H) and negative (E) charges.^38^ *In vitro* assays revealed that the HE-containing probes bound their targets in a manner similar to their parental tracers, while *in vivo* PET imaging studies in murine models of prostate cancer revealed that the charged linkers significantly reduced the kidney and spleen uptake without sacrificing accretion in tumor tissue.

#### Squaramide-Containing Linkers

Squaric acids and squaramides blur the line somewhat between linkers and bioconjugation tools (Figure 5). While squaric acid diesters have been leveraged for bioconjugation due to their ability to react sequentially with primary amines at two different pH values—i.e., the first ester will undergo amidation at pH ∼ 7, the second at pH ∼ 9—the squaramide moiety that remains in the linker *after* conjugation can also play a (minor) functional role in the radiopharmaceutical. For example, the synthon has become especially popular in the construction of ^89^Zr-labeled probes, as the two oxygens of the squaramide are hypothesized to help coordinate and stabilize the oxophilic radiometal.^39–43^ Liapis et al., for example, used a squaric ester-bearing variant of DFO to create a lupus-associated antigen-targeting immunoPET probe (i.e., [^89^Zr]­Zr-DFOsq-chDAP4) that showed promise in patients with metastatic lung cancer (Figure 5).^44^ For an excellent discussion of squaramide-containing radiopharmaceuticals, we recommend the reader refer to a recent review by Grus et al.^45^

**Figure 5:**
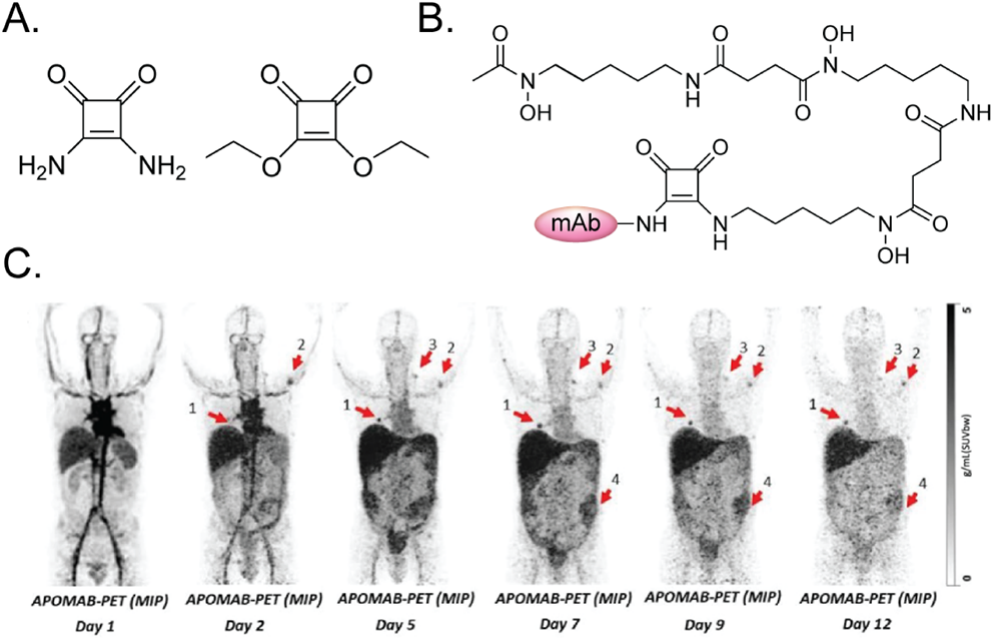
(A) Squaramide (left) and squaric acid diethyl ester (right); (B) schematic of a variant of DFO (i.e., DFOsq) conjugated to an mAb (pink oval) via a squaramide; and (C) coronal PET images acquired 1, 2, 5, 7, 9, and 12 d after the administration of the lupus-associated antigen-targeting radioimmunoconjugate [^89^Zr]­Zr-DFOsq-chDAP4 to a patient with metastatic lung cancer. Numbers and arrows denote the sites of the lesions. Adapted and reprinted from ref^44^ under a Creative Commons License (CC BY 4.0).

### Albumin-Binding Linkers

Up until this point, the static linkers that we have discussed have altered the pharmacokinetic profiles of radiotracers by modulating their *nonspecific* interactions with elements within the biological milieu. In stark contrast, the next class of static linkers is designed to improve the *in vivo* performance of radiopharmaceuticals by interacting with a *specific* protein: serum albumin. Human serum albumin is the most abundant protein in human plasma, with a concentration of ∼35–50 mg/mL (50–60% of all blood proteins). Its biological half-life is ∼19 d, both because its binding to the neonatal Fc receptor protects it from lysosomal degradation and because its high molecular weight (i.e., ∼67 kDa) precludes glomerular filtration.^46^ In light of these properties, serum albumin has attracted attention as a “molecular ferry” that can extend the blood residence time of targeted pharmaceuticals, including insulin detemir, liraglutide, and nab-paclitaxel.^47–49^ This approach is particularly relevant to nuclear medicine: binding to serum albumin offers a potent strategy to increase the biological half-life and target uptake of small molecule- and peptide-based radiopharmaceuticals, thereby boosting the contrast of imaging agents and the efficacy of therapeutics.^50^ Several different moieties have been incorporated into linkers to facilitate the covalent or noncovalent binding of radiopharmaceuticals to serum albumin, including maleimides, palmitic acid, ibuprofen, 4-(*p*-iodophenyl)­butyric acid, Evans Blue, and albumin-binding domains (ABD) (Figure 6). We will address each briefly below. For a far more comprehensive—though admittedly quite dense—discussion of the use of albumin binders in radiopharmaceutical chemistry, we recommend a recent review by Rahmati et al.^51^

**Figure 6:**
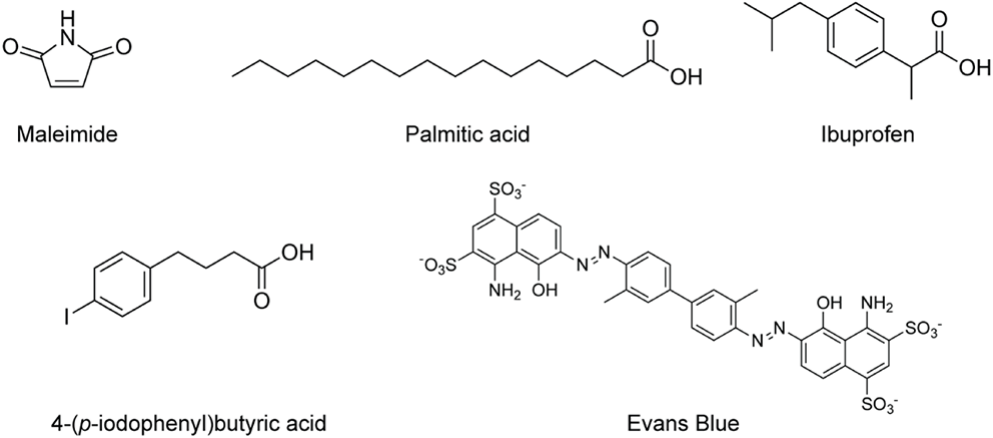
Structures of albumin-binding moieties.

#### Maleimides

Maleimide groups incorporated within radiopharmaceuticals can facilitate covalent binding to circulating serum albumin via Michael additions with the sulfhydryl groups of cysteine residues within the protein. Reports of this approach to manipulating pharmacokinetic profiles span over four decades, with Umemoto et al. describing the *in vivo* behavior of a maleimide-bearing antibody fragment-mitomycin conjugate in 1984.^52^ Far more recently, Daum et al. synthesized a variant of [^111^In]­In-DTPA modified with a pendant maleimide and demonstrated that the probe effectively radiolabeled albumin in both mouse and human serum.^53^ Subsequent SPECT imaging experiments in tumor-bearing mice revealed that the maleimide-bearing chelator produced significantly elevated activity concentration ratios in many tissues (including tumors) compared to [^111^In]­In-DTPA alone and exhibited a pharmacokinetic profile that mirrored that of exogenously radiolabeled albumin. In 2024, Feng et al. created a ^177^Lu-labeled FAP inhibitor with a linker containing a pendant maleimide, confirmed its ability to bind serum albumin, and demonstrated its *in vivo* uptake in FAP-expressing xenografts.^54^ Surprisingly, however, this work does not contain *any* comparative studies with maleimide-lacking probes, a lapse of judgment by the authors, and a misstep by the reviewers. In the end, while this strategy facilitates the covalent attachment of radiopharmaceuticals to serum albumin, its inelegance and lack of specificity—i.e., the cysteines of *any* serum protein could react with the maleimides—ultimately render it inferior to the other approaches discussed in this section.

#### Palmitic Acid

Serum albumin has several binding sites for fatty acids. Among these, the 16-carbon palmitic acid has attracted particular attention, as it has been shown to bind the protein via two high affinity sites and stabilize its native conformation.^55,56^ Conjugated fatty acids have been used to extend the half-lives of several nonradioactive pharmaceuticals—including the long-acting insulin mimetic detemir and the GLP-1 inhibitors liraglutide and semaglutide—but the benefits of albumin binding must be balanced against the increased lipophilicity conferred by the aliphatic chains.^57^ In 2022, Yang et al. synthesized a ^177^Lu-labeled radiotherapeutic containing a pair of α_v_β_3_-targeting c­(RGDyK) peptides joined via a linker containing a pendant palmitic acid (Figure 7).^58^ Compared to an analogue without the fatty acid, this albumin-binding probe exhibited a dramatically increased serum half-life and yielded increased activity concentrations in tumor tissue as well as other healthy tissues. Subsequent longitudinal studies suggest that the albumin-binding agent is a far more effective therapeutic, but these experiments were performed with equivalent doses of lutetium-177 (i.e., 18.5 MBq) rather than doses adjusted for the dosimetry of the compounds. The same year, Zhang et al. created a pair of ^68^Ga- and ^177^Lu-labeled FAP inhibitors with linkers containing C12- and C16-fatty acids and demonstrated that the latter exhibited dramatically longer biological residence time and tumoral uptake than the former.^59^ However, the lack of comparative studies with analogous FAP inhibitors without pendant aliphatic chains limits the inferences that can be made from this work.

**Figure 7:**
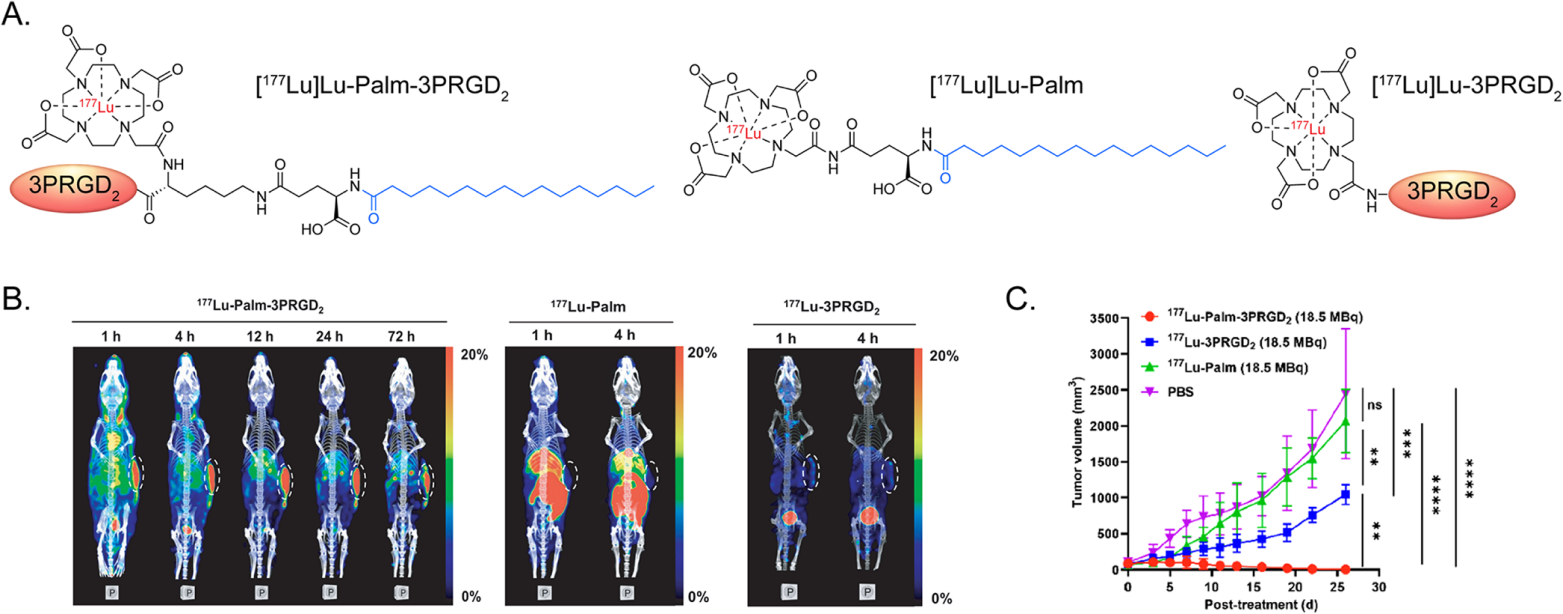
(A) Structures of [^177^Lu]­Lu-Palm-3PRGD_2_, [^177^Lu]­Lu-Palm, and [^177^Lu]­Lu-3PRGD_2_ with palmitic acid groups in blue. (B) Representative SPECT/CT images of mice bearing MC38 xenografts following the administration of [^177^Lu]­Lu-Palm-3PRGD_2_, [^177^Lu]­Lu-Palm, and [^177^Lu]­Lu-3PRGD_2_. (C) Tumor growth curves from mice bearing MC38 tumors administered PBS (purple), [^177^Lu]­Lu-Palm-3PRGD_2_ (18.5 MBq, red), [^177^Lu]­Lu-Palm (18.5 MBq, green), and [^177^Lu]­Lu-3PRGD_2_ (18.5 MBq, blue). Adapted and reprinted from ref^58^ under a Creative Commons License (CC BY 4.0).

#### Ibuprofen

Ibuprofen can bind to serum albumin with high affinity via hydrogen bonding and hydrophobic interactions and has been used preclinically to extend the half-life of nitric oxide donors for the treatment of pancreatic cancer.^60^ To date, there is only one extant report of a radiopharmaceutical containing an ibuprofen moiety to facilitate binding to albumin. In 2022, Boinapally et al. constructed a series of ^177^Lu-labeled PSMA inhibitors with an ibuprofen moiety incorporated into the linker bridging the chelator and warhead (Figure 8).^61^ *In vivo* biodistribution data collected using a murine model of prostate cancer clearly revealed that the ibuprofen extended the serum half-life of the probes and increased their uptake in both target *and* nontarget tissues; furthermore, the data showed that ibuprofen exerted a pharmacokinetic influence similar to that of another albumin-binding small molecule, 4-(*p*-iodophenyl)­butyric acid (*vide infra*). However, all of the aforementioned radiotherapeutics exhibited tumor-to-healthy organ activity concentration ratios well below those achieved by an analogous probe without an albumin-binding group.

**Figure 8:**
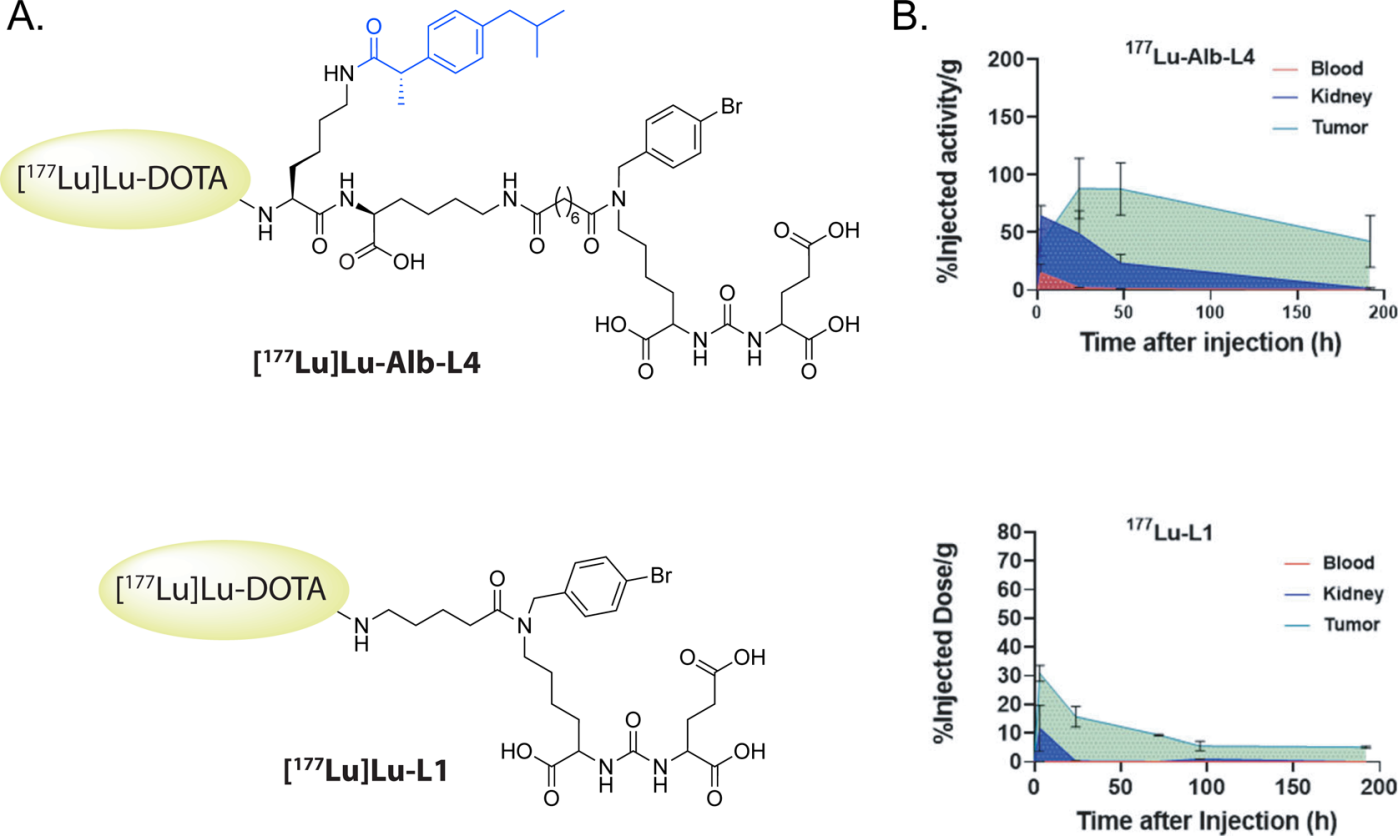
(A) Structures of [^177^Lu]­Lu-Alb-L4 and [^177^Lu]­Lu-L1 with the ibuprofen moiety in blue. (B) Comparison of the tumor, blood, and kidney time-activity curves created by the two ^177^Lu-labeled radiotherapeutics. Adapted and reprinted from ref^61^ under a Creative Commons License (CC BY 4.0).

#### Albumin-Binding Domains (ABDs)

Albumin-binding domains (ABDs) are small, stable, three-helix motifs typically found within the surface proteins of Gram-positive bacteria (e.g., streptococcal protein G and protein PAB from *Finegoldia magna*) that increase bacterial virulence by extending serum half-life and promoting immune evasion (Figure 9).^62^ ABDs have been conjugated and fused to proteinaceous drugs to extend their biological residence times, and recent work has even sought to create bispecific proteins by building antigen-specific binding sites directly into ABDs [i.e., albumin-derived affinity proteins (ADAPTs)]. These albumin binders are—of course—not linkers *per se*, but we have chosen to include them here for the sake of completeness.

**Figure 9:**
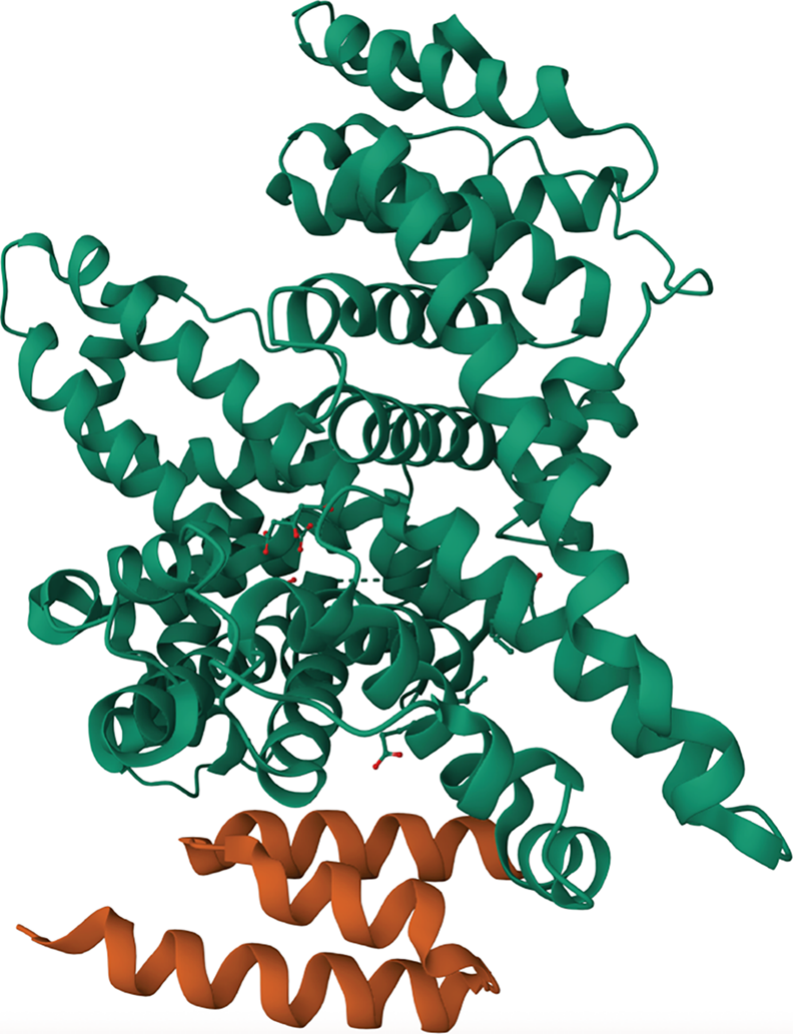
Ribbon structure of an albumin-binding domain (ABD, orange) bound to human serum albumin (HSA, green). The structure was rendered from PDB ID: 1TF0.

Within nuclear medicine, the most important and innovative work with ABDs has emerged from Vladimir Tolmachev’s laboratory at Uppsala University in Sweden. Over the past decade, the team has developed a family of fusion proteins—most notably ABY-027—composed of HER2-targeting affibody molecules and ABDs, and ^177^Lu- and ^111^In-labeled variants of these probes have shown superior *in vivo* performance in murine models of HER2-expressing cancer compared to affibody molecules without ABDs (Figure 10).^63^ More recently, the same laboratory developed G3-ABD, a construct in which a HER2-targeting designed ankyrin repeat protein (DARPin) is fused to an ABD.^64^ *In vivo* biodistribution and SPECT imaging experiments in a mouse model of HER2-positive ovarian cancer demonstrated that [^177^Lu]­Lu-G3-ABD exhibited a longer serum half-life, substantially higher tumor uptake, and dramatically lower kidney retention than a [^177^Lu]­Lu-labeled variant of the unmodified DARPin. However, comparative experiments with [^177^Lu]­Lu-ABY-027 suggested that the affibody molecule-based radiotherapeutic boasted better *in vivo* performance overall than its DARPin-based cousin.

**Figure 10:**
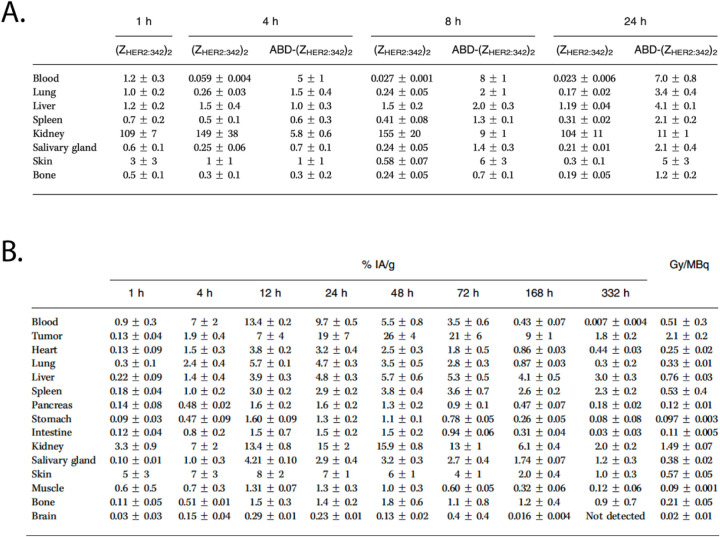
Biodistribution data for a dimeric HER2-specific affibody—(Z_HER2:342_)_2_—and a dimeric HER2-specific affibody bearing an albumin-binding domain—ABD-(Z_HER2:342_)_2_—in (A) healthy mice and (B) mice bearing HER2-positive SKOV-3 xenografts. Adapted and reprinted from ref^63^ under a Creative Commons License (CC BY 4.0).

Shifting laboratories, Hu et al. reported the development and *in vivo* validation of a trio of claudin 18.2-targeted ^89^Zr-labeled radioimmunoconjugates: a V_HH_, a V_HH_-ABD fusion protein, and a dimeric V_HH_-Fc fusion protein (Figure 11).^65^ PET imaging and biodistribution experiments in mice bearing CO-SNU620 gastric cancer xenografts demonstrated that the [^89^Zr]­Zr-V_HH_-ABD construct exhibited markedly improved tumor uptake and tumor-to-background activity concentration ratios compared to the [^89^Zr]­Zr-V_HH_; however, the *in vivo* performance of the former was superseded by that of the dimeric [^89^Zr]­Zr-V_HH_-Fc immunoglobulin (Figure 11). Ultimately, ABDs clearly offer an effective tool for modulating the albumin binding (and thus pharmacokinetics) of radioconjugates, but it is important to note that their applications will generally be limited to larger vectors, such as antibody fragments and antibody mimetics.

**Figure 11:**
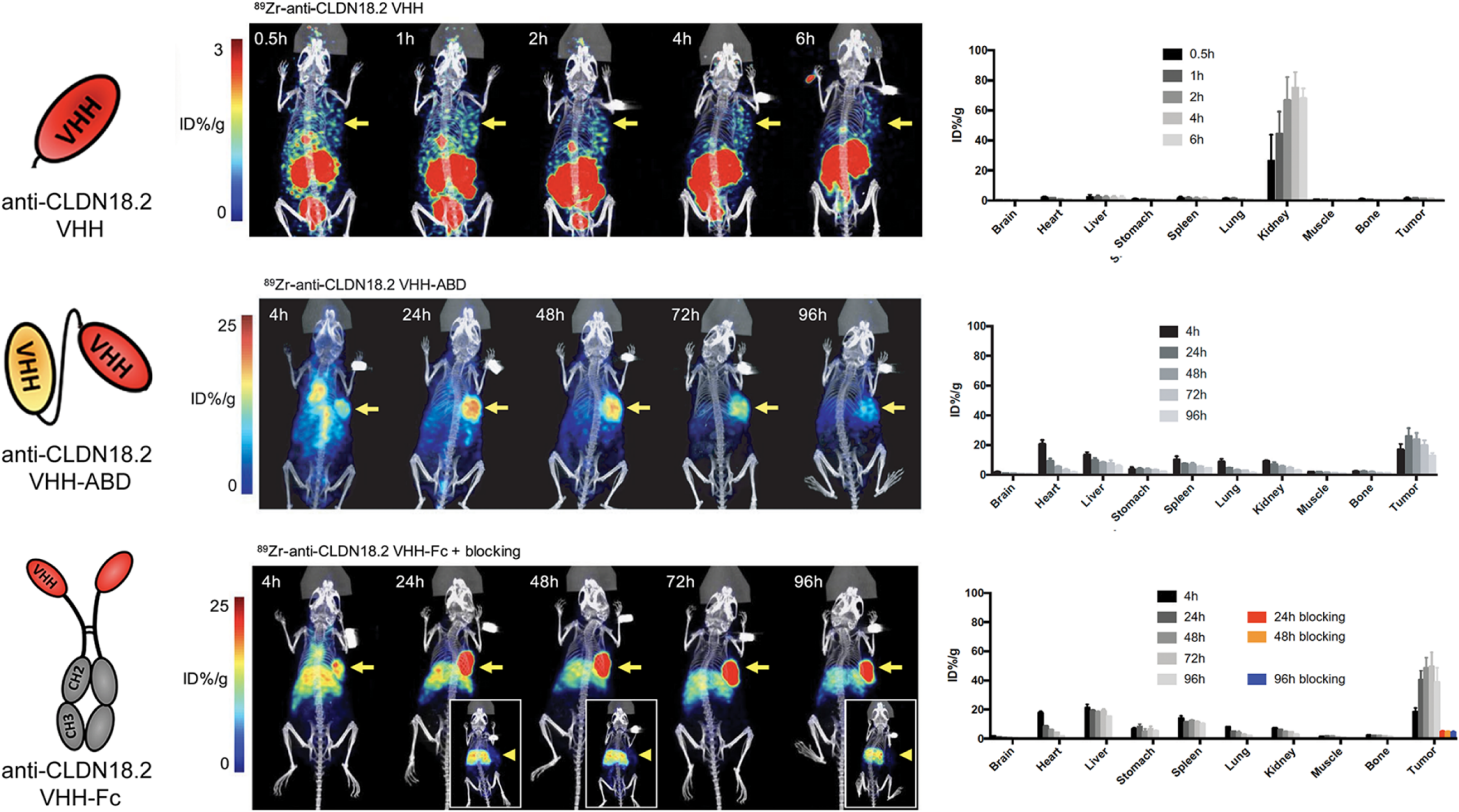
(Left) Schematics of anti-CLDN18.2-V_HH_, anti-CLDN18.2-V_HH_-ABD, and anti-CLDN18.2-V_HH_-Fc. (Center) Longitudinal PET/CT images acquired after the administration of ^89^Zr-labeled variants of each construct to mice bearing subcutaneous CO-SNU620 xenografts. (Right) Biodistribution data acquired after the administration of ^89^Zr-labeled variants of each construct to mice bearing subcutaneous CO-SNU620 xenografts. Adapted and reprinted with permission from ref^65^.

#### Evans Blue

Evans Blue (EB) is an anionic azo dye with high affinity for human serum albumin discovered over a century ago.^66^ In the ensuing years, it has been attached to a variety of constructs—including exendin-4, RGD, and an anti-tissue factor Fab—to increase their blood half-lives, but it has attracted particular attention in radiopharmaceutical chemistry. In 2017, for example, Chen et al. created ^90^Y- and ^64^Cu-labeled c­(RGDyK) agents with linkers containing a pendant EB moiety (i.e., [^64^Cu]­Cu-NMEB-RGD and [^90^Y]­Y-DMEB-RGD) (Figure 12).^67^ PET imaging experiments in a murine model of glioblastoma revealed that [^64^Cu]­Cu-NMEB-RGD produced higher activity concentrations in the tumor (16.6 ± 2.0 %ID/g) and blood (2.5 ± 0.2 %ID/g) after 24 h than an analogous construct without the albumin binder (<1.0 and <0.2 %ID/g, respectively). Companion longitudinal therapy experiments demonstrated that [^90^Y]­Y-DMEB-RGD exhibited a dose-dependent therapeutic effect that was significantly improved compared to that of [^90^Y]­Y-DOTA-RGD without the albumin binder. More recently, Wen et al. developed a series of ^177^Lu-labeled FAP inhibitors in which an Evans Blue moiety is incorporated within a PEG linker between the pyrrolidine warhead and the chelator.^68^ *In vitro* assays confirmed that these probes exhibited high affinity for both FAP and serum albumin, and SPECT imaging and biodistribution experiments in mice bearing U87MG xenografts demonstrated that they produced high tumoral uptake and one (i.e., the variant without any ethylene oxide units in its linker) exerted a dose-dependent therapeutic effect. While the investigators clearly demonstrated that one of the EB-bearing agents exhibited significantly prolonged blood residence times compared to an analogue without EB, no comparative imaging or biodistribution studies were performed in tumor-bearing mice.

**Figure 12:**
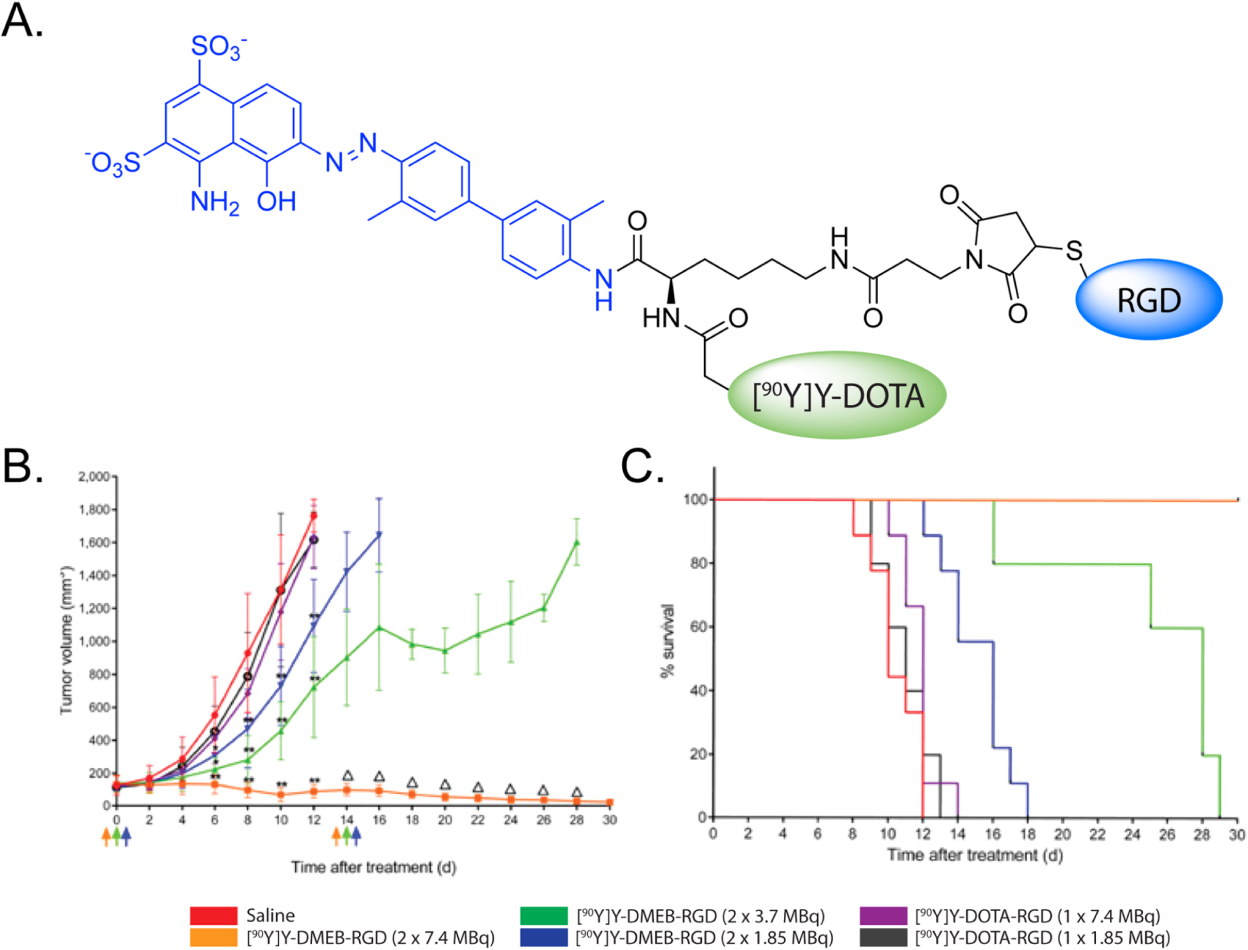
(A) Structure of [^90^Y]­Y-DMEB-RGD; (B) graphs of tumor volume over time; and (C) median survival from a longitudinal therapy study in which mice bearing U87MG xenografts were administered saline, [^90^Y]­Y-DMEB-RGD (2 × 7.4 MBq; 2 × 3.7 MBq; 2 × 1.85 MBq), or [^90^Y]­Y-RGD (2 × 7.4 MBq; 2 × 3.7 MBq; 2 × 1.85 MBq). Adapted and reprinted from ref^67^ under a Creative Commons License (CC BY 4.0).

In recent years, this technology has reached the clinic. In 2021, Hänscheid et al. performed intraindividual comparisons of [^177^Lu]­Lu-DOTA-TOC and an Evans Blue-bearing variant of the agent (i.e., [^177^Lu]­Lu-EB-TATE) in 5 patients with SSTR-expressing tumors.^69^ The data revealed that the tumor doses per unit activity were higher for [^177^Lu]­Lu-EB-TATE in 4/5 patients; however, the absorbed doses to the kidneys and spleen were higher in 5/5 patients, and the dose to the liver was higher in 3/5. Furthermore, the tumor-to-critical organ absorbed dose ratios were higher for [^177^Lu]­Lu-DOTA-TOC in 4/5 patients. In 2024, Guo and workers developed another EB-bearing SSTR2-targeting radiotherapeutic—[^177^Lu]­Lu-LNC1010—and performed both preclinical and clinical studies comparing it to [^177^Lu]­Lu-EB-TATE.^70^ SPECT and biodistribution studies in mice bearing AR42J expressing xenografts revealed that the former radiotherapeutic broadly outperformed the latter. A subsequent clinical trial in 12 patients with SSTR-expressing tumors revealed that [^177^Lu]­Lu-LNC1010 was well tolerated and produced an ∼80% disease control rate and ∼40% overall response rate after two cycles; comparisons with three patients treated with [^177^Lu]­Lu-EB-TATE suggested that the former produced a higher absorbed dose to the tumor than the latter (Figure 13). Regrettably, no preclinical or clinical comparisons to [^177^Lu]­Lu-DOTA-TATE or [^177^Lu]­Lu-DOTA-TOC were included.

**Figure 13:**
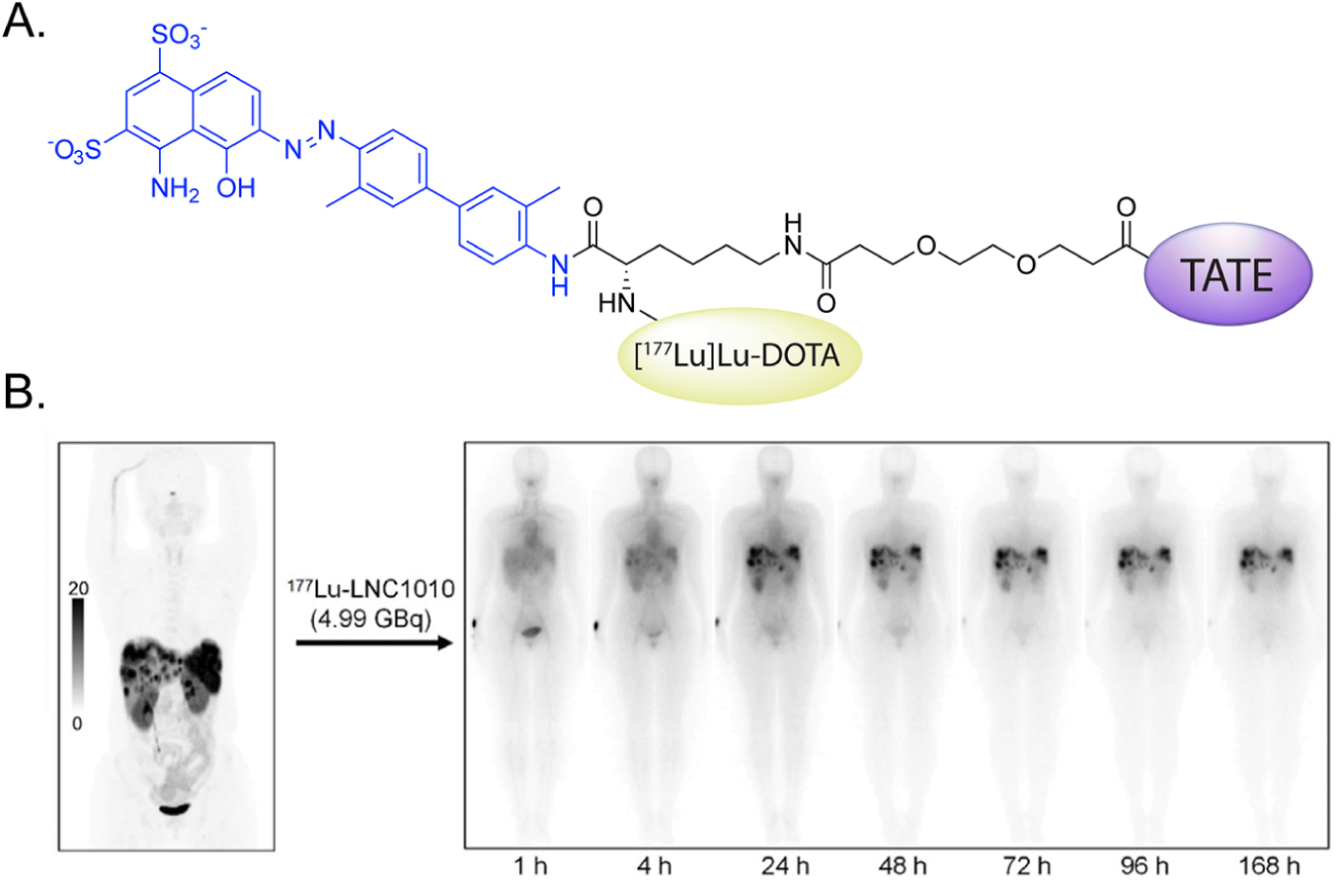
(A) Structure of [^177^Lu]­Lu-LNC1010 with the Evans Blue moiety in blue. (B) Pretherapeutic [^68^Ga]­Ga-DOTA-TATE PET/CT scan of a 37-year-old patient with metastatic pancreatic NET, as well as whole-body scintigraphy images of the same patient acquired at several time points after the administration of [^177^Lu]­Lu-LNC1010. Adapted and reprinted from ref^70^ under a Creative Commons License (CC BY-NC-ND 4.0).

#### 4-(*p*-Iodophenyl)­butyric Acid

Derivatives of butyric acid bearing iodophenyl moieties—especially 4-(*p*-iodophenyl)­butyric acid (IPBA)—bind to hydrophobic pockets within serum albumin with high affinity and thus have been widely used to extend the serum half-life of pharmaceuticals.^71,72^ Indeed, IPBA and its relatives are far and away the most commonly used serum albumin binders in nuclear medicine, with nearly two dozen different reports of radiopharmaceuticals in which it is incorporated. In 2018, for example, Benesova et al. developed a trio of ^177^Lu-labeled variants of PSMA-617 containing linkers with pendant IPBA groups.^73^ In murine models of prostate cancer, all three probes exhibited longer plasma half-lives, elevated tumoral accretion, and higher tumoral area-under-the-curve (AUC) values; however, [^177^Lu]­Lu-PSMA-617 outperformed all of the IPBA-bearing variants in terms of tumor-to-blood, tumor-to-liver, and tumor-to-kidney AUC ratios. A year earlier, another team used click chemistry to assemble ^177^Lu-labeled phosphoramidate-based PSMA inhibitors with ([^177^Lu]­Lu-CTT1403) and without ([^177^Lu]­Lu-CTT1401) linkers containing IPBA moieties (Figure 14).^74^ *In vivo* biodistribution studies in mice bearing PSMA-expressing PC3 xenografts demonstrated that [^177^Lu]­Lu-CTT1403 boasted significantly higher activity concentrations in the tumor, blood, and kidneys over time; furthermore, while [^177^Lu]­Lu-CTT1403 provided good tumor-to-kidney and tumor-to-muscle activity concentration ratios (particularly at later time points), [^177^Lu]­Lu-CTT1401 provided substantially improved tumor-to-blood ratios across all time points. Finally, longitudinal therapy studies in the same murine model of disease revealed that [^177^Lu]­Lu-CTT1403 was significantly more effective than [^177^Lu]­Lu-CTT1401, as treatment with the former produced substantially better tumor control and a greater median overall survival.

**Figure 14:**
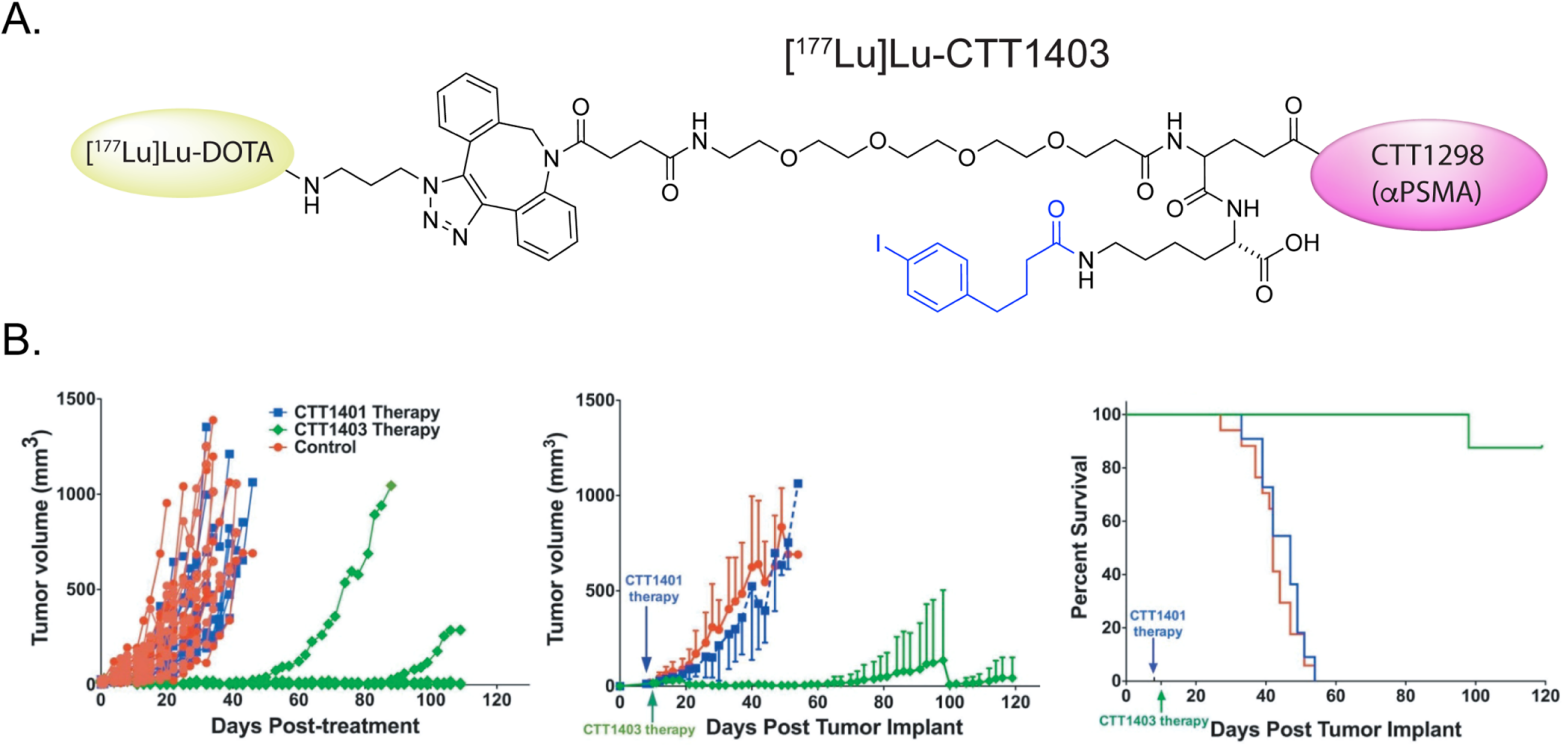
(A) Structure of [^177^Lu]­Lu-CTT1403; (B) graphs of individual tumor volume, mean tumor volume, and median survival from a longitudinal therapy study in which NCr mice bearing PC3-PIP xenografts were administered saline, [^177^Lu]­Lu-CTT1403 (0.37 MBq), and [^177^Lu]­Lu-CTT1401 (0.37 MBq). Adapted and reprinted from ref^74^ under a Creative Commons License (CC BY-NC 4.0).

Shifting to other targets, Muller et al. used copper-catalyzed azide-alkyne click chemistry to assemble [^177^Lu]­Lu-cm09, a folate receptor-targeting probe containing a linker with a tethered IPBA group.^75^ SPECT imaging experiments in mice bearing subcutaneous KB tumors clearly showed that [^177^Lu]­Lu-cm09 produced a higher tumor uptake and lower kidney retention than [^177^Lu]­Lu-EC0800, an analogous compound without the IPBA moiety. Subsequent studies established the therapeutic efficacy of [^177^Lu]­Lu-cm09, but—unfortunately—no comparative studies with [^177^Lu]­Lu-EC0800 were shown. In a similar vein, Iikuni et al. created a pair of ^111^In-labeled variants of the GLP-1R-targeting peptide exendin, with and without a pendant IPBA group, and used SPECT imaging in a murine model of insulinoma to illustrate that the former produced greater tumoral uptake, lower kidney retention, and substantially superior tumor-to-kidney activity concentration ratios.^76^ Finally, just a few years ago, Julie Sutcliffe’s team at the University of California Davis created α_v_β_6_-targeting ^68^Ga- and ^177^Lu-labeled DOTA agents containing linkers with IPBA moieties.^77^ PET imaging in mice inoculated with BxPC-3 xenografts demonstrated that the IPBA-containing ^68^Ga-labeled probe produced higher uptake in the tumor and blood as well as higher tumor-to-kidney activity concentration ratios than its analogue, which cannot bind albumin, though the latter yielded higher tumor-to-blood, -liver, and -pancreas activity concentration ratios. Biodistribution experiments with the ^177^Lu-labeled agents provided more compelling results in light of their longer time course: here, the albumin-binding variant provided higher tumor accretion and tumor-to-blood, -stomach, and -pancreas activity concentration ratios. Longitudinal therapy experiments clearly demonstrated the efficacy of the IPBA-bearing ^177^Lu-labeled agent, but no comparative experiments were described.

Taken together, these studies clearly demonstrate the effectiveness of IPBA as an albumin binder and pharmacokinetic modulator. That said, it is important to note that their greatest utility seems to lie in situations in which higher degrees of *absolute* uptake in target tissue are needed, as the higher target accretion that comes with albumin binding is often (but not always) accompanied by elevated uptake in healthy tissues and thus lowered target-to-healthy tissue activity concentration ratios.

## Dynamic Linkers

As we have discussed, the most fundamental job of a linker is to ensure that the vector and the radionuclide remain connected during the radiopharmaceutical’s transit through the body. However, there are some circumstances in which it may be advantageous for the two components to decouple within the body. For example, the release of the radiometal-chelator complex from a macromolecular probe sequestered in a nontarget tissue could facilitate the rapid excretion of the far smaller former moiety and thus could reduce off-target radiation dose rates. In light of this, several strategies have been developed to facilitate the spatially and/or temporally controlled cleavage of linkers within radiopharmaceuticals (Figure 15). Generally speaking, these approaches fall into two subclasses based on whether the origin of the cleavage-initiating stimulus is endogenous or exogenous; we will discuss both here.

**Figure 15:**
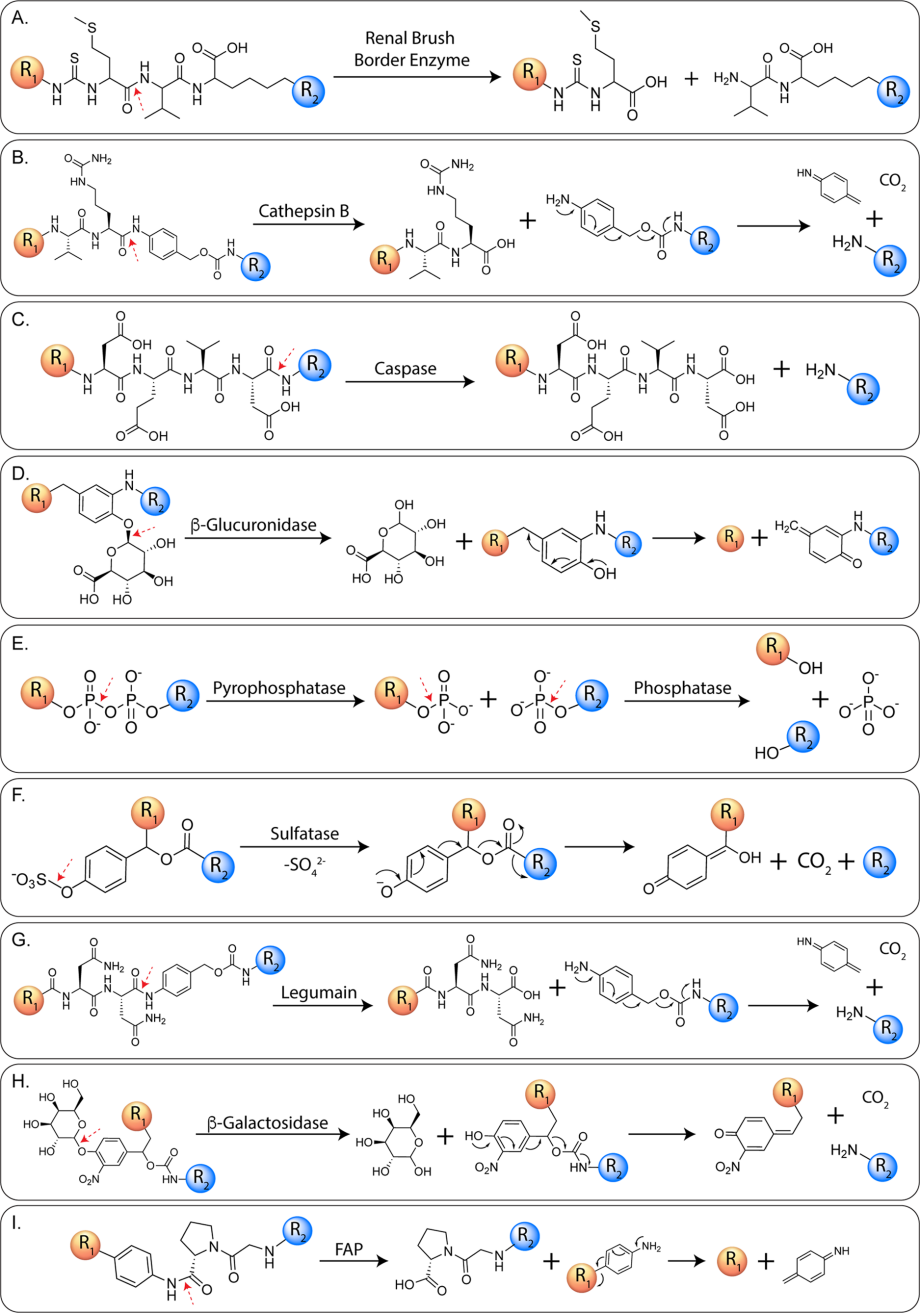
Schematics illustrating the cleavage of linkers by endogenous (A) renal brush border enzyme, (B) cathepsin B, (C) caspase, (D) β-glucuronidase, (E) pyrophosphatase and phosphatase, (F) sulfatase, (G) legumain, (H) β-galactosidase, and (I) fibroblast activation protein. R_1_ and R_2_ represent the payload; red arrows denote the site of enzymatic cleavage.

### Linkers Cleaved by Endogenous Stimuli

The use of conjugates bearing linkers that can be cleaved by endogenous stimuli can be advantageous if the separation of the vector and payload in specific tissues can increase efficacy or decrease toxicity. This strategy is particularly attractive in the context of antibody-drug conjugates, as the decoupling of the toxin and immunoglobulin can facilitate cellular penetration of the drug, especially when the antibody is not rapidly internalized. Indeed, as of 2025, 13 of 17 FDA-approved ADCs employ cleavable linkers.^78^ It is important to note at the outset of this section that these linkers are markedly less popular in nuclear medicine. This difference largely stems from the fundamentally distinct mechanisms of these drugs. In the context of ADCs, *in vivo* cleavage of the toxin can dramatically boost efficacy by facilitating the cellular uptake of the drug, which is essential to its cytotoxicity. However, in the context of radiopharmaceuticals, the *in vivo* decoupling of the radionuclide and vector is not strictly necessary for effective imaging or therapy, and the consequences of this release—i.e., the redistribution of the radionuclide to healthy tissues and their subsequent irradiation—can be dire. Nonetheless, radiopharmaceutical chemists have found ways to exploit cleavable linkers, most notably to facilitate the clearance of radionuclides from healthy tissues in which radiolabeled compounds have accumulated.

Cleavable linkers are designed with two key factors in mind: they must be stable—both in the body or on the shelf—in the absence of their designated chemical trigger while simultaneously releasing their payload specifically and rapidly under the desired *in vivo* conditions.^78–80^ These linkers are typically cleaved by one of two types of endogenous stimuli: enzymes (e.g., cathepsins, caspases, etc.) and small molecules (e.g., glutathione, reactive oxygen species, pH). We will address each of these types of linkers in the following paragraphs.

### Enzymatically Cleaved Linkers

#### Renal Brush Border Enzymes

Small molecules and peptides are attractive platforms for radiopharmaceuticals due to their rapid pharmacokinetic profiles, but one common limitation of these agents is their uptake and retention in the kidneys. The same can be said of small immunoglobulins, such as single domain antibodies (sdAb) and single chain variable fragments (scFv), which offer comparable target specificity and affinity to full-length antibodies but more rapid pharmacokinetic profiles. While in both cases this pharmacokinetic behavior can reduce tumor-to-background contrast during nuclear imaging, it is particularly problematic in radiopharmaceutical therapy, as the kidneys are especially radiosensitive tissues. A variety of approaches have been explored to mitigate this issue.^81–87^ However, the most germane to the topic at hand is the use of radiopharmaceuticals bearing linkers that can be cleaved by renal brush border enzymes (RBBEs), a family of enzymes—including neprilysin, carboxypeptidase M, and other amino- and endopeptidases—expressed in the microvilli of kidney tubule cells (Figure 15A).^81–83^ The concept here is straightforward: once a radiopharmaceutical is sequestered in the kidneys, RBBEs will cleave these linkers, liberating the radionuclide-chelator complex (or radiolabeled prosthetic group) so that it can be rapidly cleared from the body.^81–87^

A group from Chiba University has spearheaded the use of RBBE-cleavable linkers in radiopharmaceuticals. Indeed, this group has published several reports of radiopharmaceuticals with RBBE-cleavable linkers that exhibit reduced kidney retention in murine models.^84,88,89^ For example, in 2018, Uehara et al. created a [^67/68^Ga]­Ga-NOTA-labeled variant of a c-kit-targeting Fab with an RBBE-cleavable Met-Val-Lys (MVK) linker (Figure 16).^84^ *In vivo* SPECT imaging experiments in mice bearing subcutaneous SY non-small cell lung cancer xenografts revealed that the ^67^Ga-labeled probe with the MVK linker produced images with comparable tumoral uptake, lower kidney retention, and improved tumor-to-kidney contrast compared to analogues with either no linker or a Met-Ile linker that produces different radiometabolites. That same year, a different group, led by Vaidyanathan et al., created ^131^I- and ^211^At-labeled radiopharmaceuticals featuring a PSMA-targeting Glu-ureido pharmacophore and an RBBE-cleavable linker.^71^ Subsequent experiments in mice bearing LNCaP human prostate cancer xenografts revealed that a radioiodinated PSMA-targeted tracer with an RBBE-cleavable linker exhibited dramatically reduced kidney retention and significantly increased tumor-to-kidney activity concentration ratios compared to a comparable probe without the cleavable linker. Subsequent preclinical studies from other groups confirmed these observations.^85–87^

**Figure 16:**
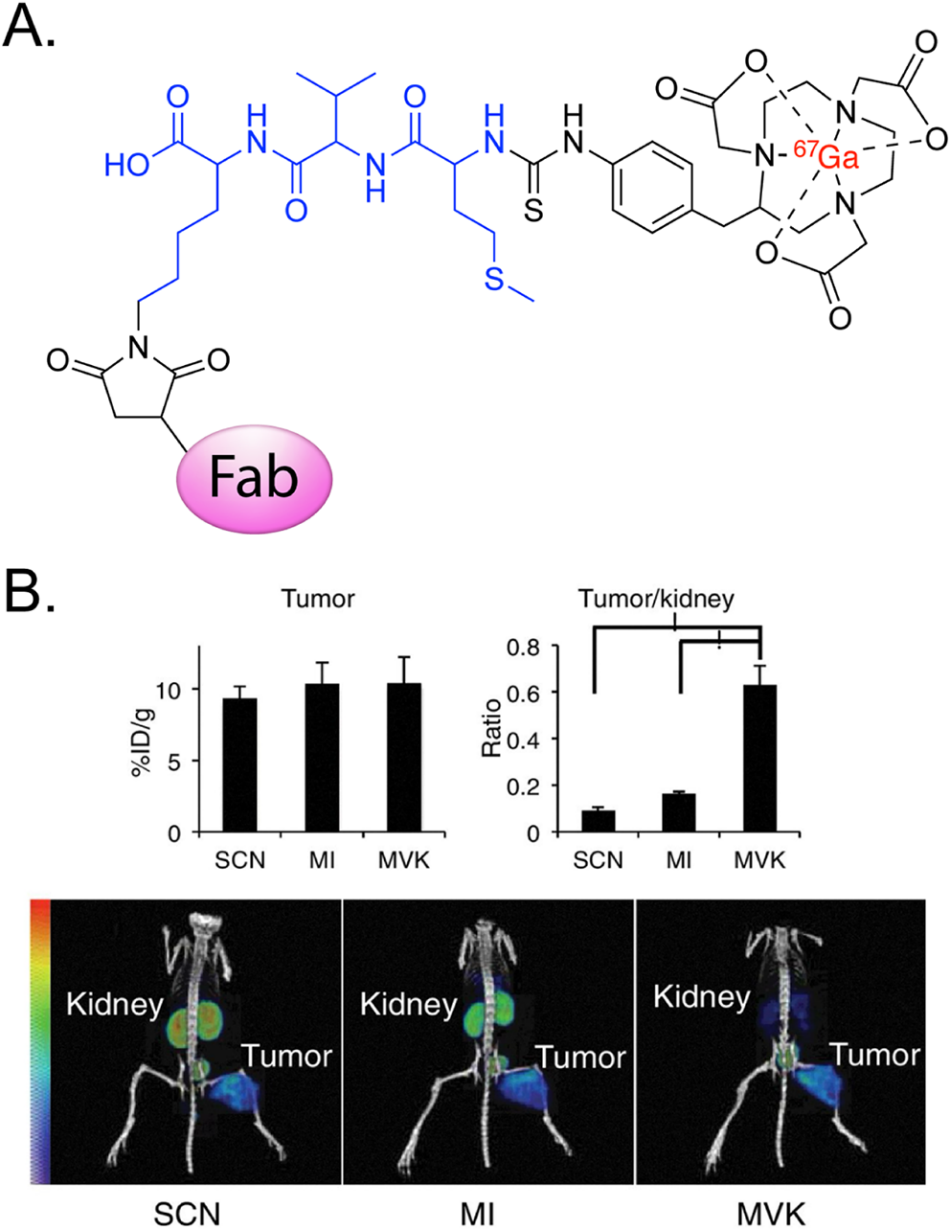
(A) Structure of the [^67^Ga]­Ga-NOTA-Fab radioimmunoconjugate with an RBBE-cleavable MVK linker developed by Uehara et al. (B) Tumoral activity concentrations (top left), tumor-to-kidney activity concentration ratios (top right), and representative SPECT/CT images (bottom) acquired following the administration of [^67^Ga]­Ga-NOTA-MVKFab (i.e., “MVK”), a ^67^Ga-labeled analogue with a methionine-isoleucine dynamic linker (i.e., “MI”), and a ^67^Ga-labeled analogue with a static linker (i.e., “SCN”) in nude mice bearing subcutaneous non-small cell lung cancer SY tumors. Adapted and reprinted with permission from ref^84^.

The first clinical trial to employ a radiopharmaceutical with an RBBE-cleavable linker was published in 2024 by Zhang et al. This work was centered on a novel HER2-targeted affibody molecule in which an MVK-based linker tethered the biomolecule to a [^68^Ga]­Ga-NOTA moiety (Figure 17).^90^ PET imaging in healthy volunteers as well as patients with HER2-positive breast or gastric cancer revealed significant decreases in the renal accumulation of the MVK linker-bearing radiotracer compared with an analogous affibody with a static linker. Critically, both the tumoral accretion and tumor-to-background activity concentration ratios remained high for the affibody with the dynamic linker.

**Figure 17:**
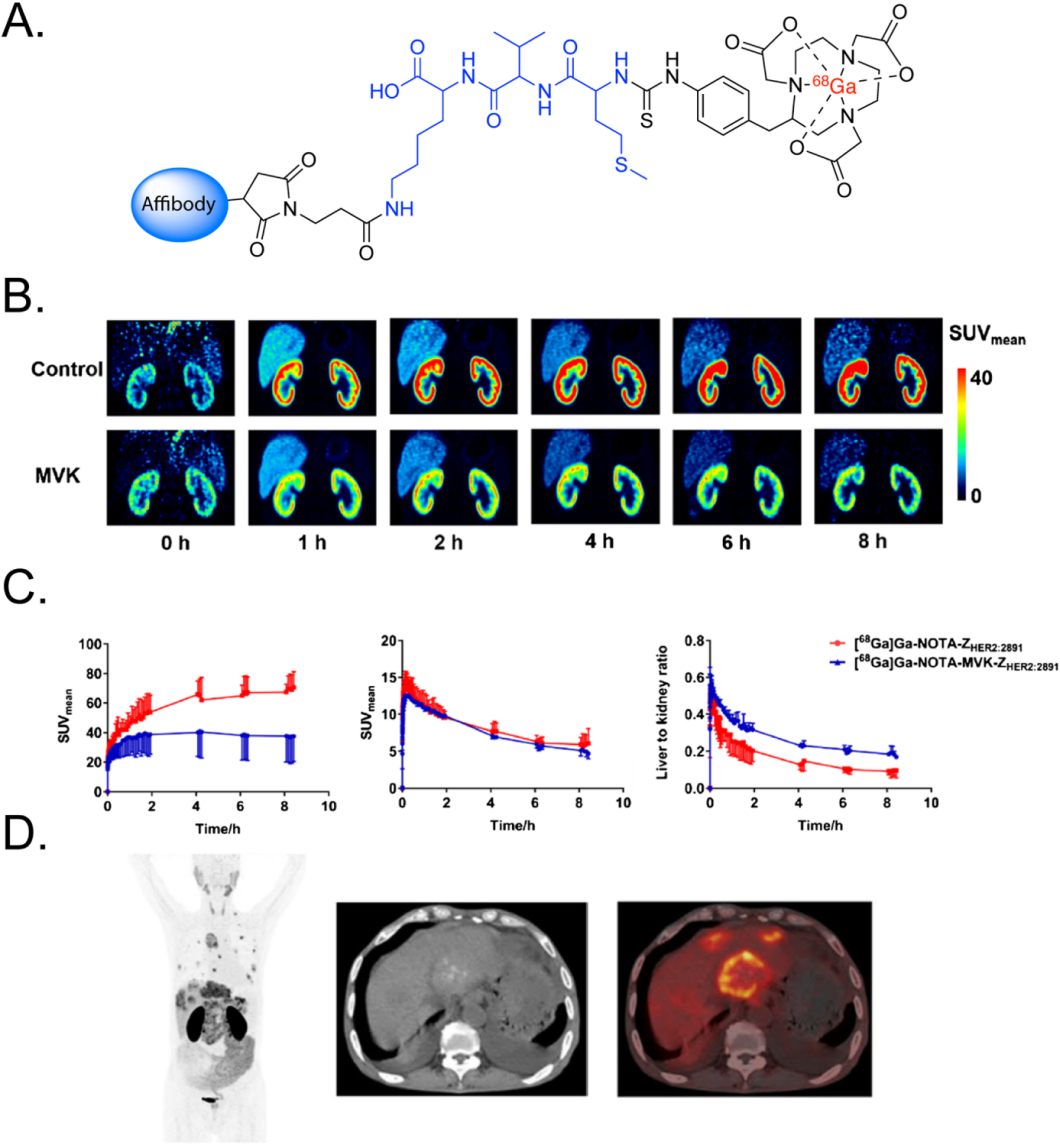
(A) Structure of a ^68^Ga-labeled affibody with an RBBE-cleavable MVK linker; (B) PET images of the kidneys acquired at various time points after the administration of [^68^Ga]­Ga-NOTA-Z_HER2:2891_ (top) and [^68^Ga]­Ga-NOTA-MVK-Z_HER2:2891_ (bottom) to a patient with breast cancer; (C) time-activity curves for the liver (left) and kidneys (middle) as well as liver-to-kidney activity concentration ratios as a function of time acquired after the administration of [^68^Ga]­Ga-NOTA-Z_HER2:2891_ (red) and [^68^Ga]­Ga-NOTA-MVK-Z_HER2:2891_ (blue) to patients (*n* = 3) with breast and gastric cancer; (D) coronal PET (left), transverse CT (center), and transverse PET/CT (right) acquired 2 h after the administration of [^68^Ga]­Ga-NOTA-MVK-Z_HER2:2891_ to a patient with gastric cancer. Adapted and reprinted with permission from ref^90^.

#### Cathepsins

Intracellular proteases are particularly popular candidates for the development of enzyme-cleavable linkers. Without question, the most popular of these proteases is cathepsin B, a lysosomal cysteine protease that cleaves dipeptide bonds. The earliest known example of the use of cathepsin B-cleavable linkers stems from 1998, when Dubowchik et al. created ADCs bearing cleavable linkers containing dipeptides such as Phe-Arg, Phe-Lys, Val-Cit, and Val-Ala as well as a para-aminobenzyl carbamate moiety that can release a form of doxorubicin.^91^ Since this work, these types of linkers have been used extensively.^78^ Indeed, a wide variety of successful ADCs—including trastuzumab deruxtecan (Enhertu), enfortumab vedotin (Padcev), and brentuximab vedotin (Adcetris)—have employed cathepsin-cleavable Val-Cit linkers (Figure 15B).

A handful of radiopharmaceuticals have been created bearing cathepsin-cleavable linkers designed to decrease the retention of radionuclides in nontarget tissues. For example, DeNardo et al. created ^111^In-labeled MUC1-targeting radioimmunoconjugates with a cathepsin-cleavable linker and found that this construct produced comparable tumoral uptake, lower hepatic retention, and greater therapeutic ratios than an analogous construct without the cleavable linker.^92^ Bolstered by these promising results, the same team translated ^111^In- and ^90^Y-labeled variants of this radioimmunoconjugate to the clinic, ultimately finding that the agents accumulated in prostate and breast cancer tumor tissue and were well tolerated.^93^ In 2006, however, a team from Memorial Sloan Kettering Cancer saw strikingly different results. Antczak et al. observed that an ^225^Ac-labeled radioimmunoconjugate bearing a cathepsin-cleavable linker conjugated via a maleimide-thiol bond produced higher uptake and prolonged liver retention compared to an analogous ^225^Ac-labeled mAb containing a noncleavable linker conjugated via a lysine-isothiocyanate coupling.^94^ Curiously, however, the biodistribution of the former was similar to that of a probe bearing a noncleavable linker conjugated via a maleimide–thiol bond, suggesting that the root of this phenomenon may lie in the bioconjugation method. Finally, Chastel et al. created a pair of ^111^In-labeled human Y1 receptor-targeting peptides that include a nuclear localization signal attached to a cathepsin-cleavable linker, a design intended to increase the uptake of the radiometal in the nucleus and thus enhance Auger electron-mediated cytotoxicity.^95^ Unfortunately, the authors found that those radioconjugates were not stable in serum or *in vivo*, ending the development effort.

#### Caspases

Caspases are a family of cytosolic proteases that play essential roles in apoptosis and have also been interrogated as possible triggers for endogenously cleaved linkers. In an effort to increase cell killing via the bystander effect, Lee et al. developed a HER2-targeting exatecan ADC held together by a caspase-3-cleavable Asp-Glu-Val-Asp (DEVD) linker (Figure 15C).^96^ The authors found that the ADC targeted tumor tissue, released its payload *in vivo* in a caspase-dependent manner, and exerted *in vivo* efficacy comparable to that of trastuzumab-deruxtecan; however, the lack of comparative control experiments with an analogous ADC without the cleavable linker makes the interpretation of these data challenging. While caspases—most notably caspase-3—have been explored as targets for PET imaging, no radiopharmaceuticals bearing caspase-cleavable linkers have been developed, likely because this “help” with the bystander effect is not needed in the context of radionuclide therapy.^97^

#### β-Glucuronidases

β-Glucuronidases (βG) are lysosomal glycosidases that catalyze the hydrolysis of β-glucuronic acid residues from polysaccharides. The enzyme can be found in microsomes (i.e., the endoplasmic reticulum) and lysosomes in healthy tissues, but extracellular levels of βG have been observed in a wide variety of pathological conditions, including rheumatoid arthritis, fibrosis, neuroinflammation, and cancer. Not surprisingly, βG has attracted attention as a trigger for enzyme-activated probes, especially because it offers a more hydrophilic alternative to Val-Cit linkers.^98^ Hamilton et al., for example, developed a CD30-targeting ADC in which tubulysin M is linked to the mAb via a βG-cleavable linker (Figure 15D).^99^ The team demonstrated that the glucuronide linker improved the stability of the ADC as well as its *in vivo* activity compared to an analogous ADC bearing a traditional protease-cleavable dipeptide linker. In another example, Anselmi et al. synthesized a peptide-drug conjugate in which the α_v_β_3_-integrin ligand c­(RGDfK) is attached to cryptophycin-55 glycinate via a hydrophilic βG-cleavable linker.^98^ The conjugate exhibited 70-fold reduced cytotoxicity compared with the free drug, underscoring the ability of the linker to limit passive cellular uptake. However, βG-mediated drug release was observed upon accumulation in tumor tissue, enabling the passive diffusion of the freed drug into neighboring cancer cells for bystander killing.

A handful of PET probes have been developed that rely upon enzyme-mediated cleavage to visualize βG activity (Figure 18).^100,101^ Two of these agents—[^124^I]­I-TrapG and [^18^F]­FEAnGa—have been shown to exhibit enzyme activity-dependent uptake in βG-expressing tissues. Strictly speaking, [^124^I]­I-TrapG and [^18^F]­FEAnGa do not fit perfectly within our broader discussion: βG is their target, and thus, their βG-cleavable components are not linkers between radionuclides and targeting vectors. That said, the efficacy of these probes suggests that βG *could* be leveraged as a trigger for enzyme-responsive radiopharmaceuticals in the future.

**Figure 18:**
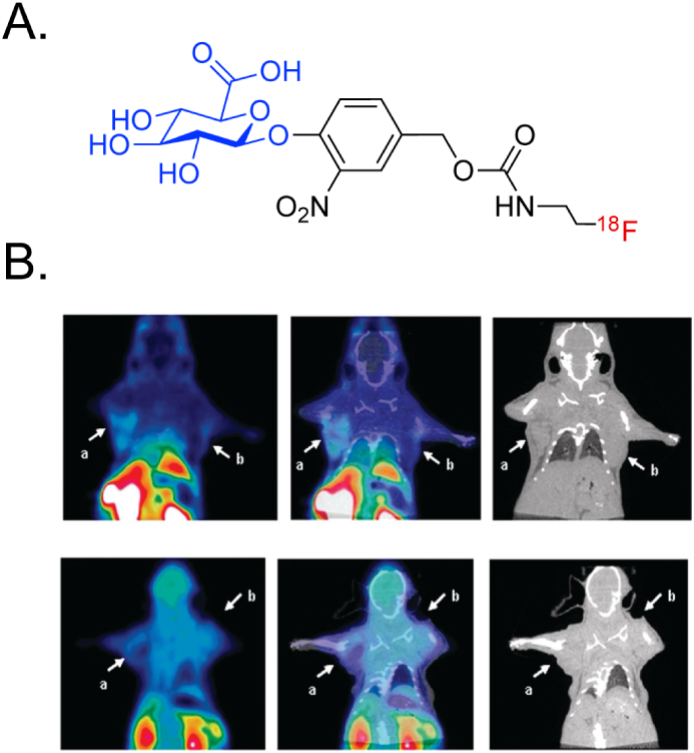
(A) Structure of [^18^F]­FEAnGa; (B) PET (left), PET/CT (middle), and CT (right) images of mice bearing bilateral CT26mβGUS (a) and CT26 (b) tumors acquired after the administration of [^18^F]­FEAnGa (top row) or [^18^F]­FEA (bottom row). Adapted and reprinted with permission from ref^101^.

#### Phosphatases and Pyrophosphatases

Phosphatases are enzymes that hydrolyze terminal monophosphates to alcohols, and the aberrant activity of some phosphatases in various pathologies (e.g., cancer) has fueled interest in harnessing them as triggers for enzymatically activated drugs. In 2023, for example, An et al. developed stimuli-responsive proteolysis targeting chimeras (PROTACs) bearing a phosphatase-sensitive linker to address issues of selectivity and efficiency associated with traditional PROTACs.^102^ These new constructs demonstrated high solubility and tumor-specific cleavage by tumor phosphatases—such as alkaline phosphatase—reducing off-target effects. In an interesting twist, Kern et al. created a hybrid strategy in which a CD70-targeting antibody was coupled to a phosphate-modified toxin via a Val-Cit-PABC linker.^103,104^ Cathepsin B-mediated cleavage of the linker could release the prodrug, after which phosphatase activity would produce the terminal payload (Figure 15E). The team demonstrated effective *in vitro* targeting, cleavage, and activation. While subsequent *in vivo* evaluations have yet to be published, Su et al. commented that the dual sensitivity of this construct may lead to its instability.^79^ In the context of nuclear medicine, PET imaging agents that target phosphatases—e.g., prostatic acid phosphatase—have been deployed in both the laboratory and clinic, but, to the best of our knowledge, there are no extant examples of radiopharmaceuticals bearing phosphatase-cleavable linkers.^105^

Along similar lines, pyrophosphatases are acid anhydride hydrolases that act upon diphosphate bonds. As pyrophosphatases are expressed within lysosomes, the enzymes (such as phosphatases) represent a potential trigger for enzyme-activated prodrugs. In one example, one of the teams discussed above (Kern et al.) created a CD70-targeting ADC bearing a diphosphate linker for the pyrophosphatase-triggered release of glucocorticoids (Figure 15E).^104^ The constructs effectively delivered the payload to CD70-expressing cells, and lysosomal release of the payload was observed; however, no *in vivo* validation was reported. While pyrophosphatases have been targeted for PET imaging (albeit rarely), there are no extant radioconjugates featuring pyrophosphatase-triggered linkers.

#### Sulfatase

Sulfatases are a class of esterases that catalyze the hydrolysis of sulfate esters. They are primarily expressed in lysosomes and the endoplasmic reticulum, but they can be found in other cellular compartments and can be secreted outside of the cell.^106,107^ The overexpression of sulfatases in some diseases—most notably cancer—makes them a potential trigger for prodrugs; this is especially true given that sulfate-containing linkers (like the phosphate-containing linkers described above) are more hydrophilic than the more commonly used Val-Cit linkages (Figure 15F). In 2020, for example, Bargh et al. developed a trastuzumab-based ADC in which MMAE is linked to the antibody via an arylsulfatase-cleavable linker.^108^ The team demonstrated the lysosomal release of the payload and found that the cleavable ADC was more potent *in vitro* than an analogous immunoconjugate without a cleavable linker. As in several of the cases above, sulfatases have been leveraged as targets for radiopharmaceuticals, but no radioconjugates with sulfatase-cleavable linkers have been reported.^109–111^

#### Legumain

Legumain is an asparaginyl endopeptidase overexpressed in several cancers, particularly within the lysosomes of cancer cells. In 2021, Miller et al. demonstrated that a trastuzumab-MMAE ADC with an Asn-Asn-PABC legumain-cleavable linker remained >85% stable in serum over 1 week (Figure 15G).^112^ Subsequent *in vivo* experiments revealed that their ADC exhibited only slightly reduced off-target effects compared to those of an analogue with a Val-Cit linker. However, they noted that legumain-cleavable linkers are not substrates for neutrophil elastase, potentially reducing neutropenia risk.

To date, no radiopharmaceuticals have been developed that employ legumain-cleavable linkers to accelerate clearance or potentiate cytotoxicity. The closest example is an ^18^F-labeled, PSMA-targeting small molecule-drug conjugate in which legumain-mediated cleavage is responsible for the release of a toll-like receptor 7 agonist (but *not* the fluorine-18).^113^ That said, several radiopharmaceuticals that depend upon enzyme-mediated cleavage for the targeting of tissues that overexpress legumain *have* been developed.^114,115^ One team from China, for example, has recently reported the creation of several ^18^F-, ^68^Ga-, and ^131^I-labeled radiopharmaceuticals that undergo macrocyclization and aggregation following “unmasking” via legumain-mediated cleavage (Figure 19).^116–118^ In murine models of cancer, the probes have exhibited selective (albeit meager) uptake in legumain-expressing xenografts compared to tumors that do not produce the enzyme.

**Figure 19:**
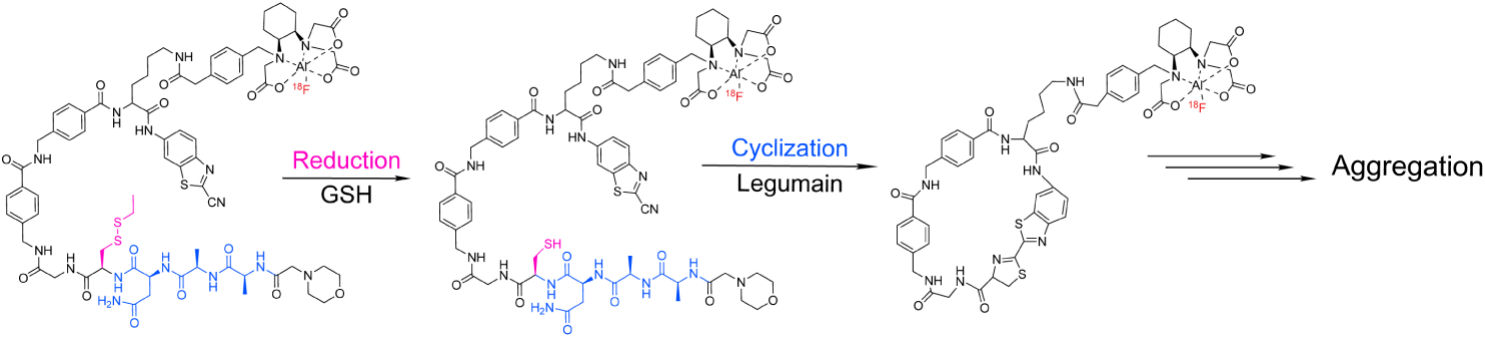
Schematic of the legumain-triggered cyclization and aggregation of [^18^F]­AlF-RSM.

#### β-Galactosidase

β-Galactosidase (β-Gal) is a glycoside hydrolase that catalyzes the hydrolysis of terminal nonreducing β-D-galactosidase residues (Figure 15H). The overexpression of β-Gal in senescent cells has long been established, leading Zhen et al. to develop a fluorogenic probe (i.e., FR-2A) in which the cleavage of a galactose-bearing self-immolative *p*-hydroxybenzyl quaternary ammonium linker by β-Gal yields a styryl-based push–pull benzothiazole fluorophore.^119^ The probe exhibited high water solubility, intense fluorescence, and a high affinity for β-Gal and proved effective for the selective imaging of senescent cells. In a similar manner, Shao et al. created galactose-capped dendrimers that can be unmasked by bacterially secreted β-Gal to yield dendrimers with antimicrobial properties.^120^

In the context of radiopharmaceutical chemistry, β-Gal has been leveraged as a tool for site-specific bioconjugation and has been targeted for PET imaging of cellular senescence. With respect to the latter, several of the radiotracers rely upon β-Gal-mediated cleavage for intracellular trapping (e.g., [^68^Ga]­Ga-BGal^121^ or [^125^I]­I-IBDG^122^) or activated self-assembly (e.g., [^68^Ga]­GaNap-NOTA-1Gal^123^) (Figure 20). However, these tracers do not necessarily fit within the remit of this review, as cleavage by β-Gal is a mechanism for targeting the enzyme itself rather than for modulating the pharmacokinetic profile of a radiopharmaceutical targeting some other species.

**Figure 20:**
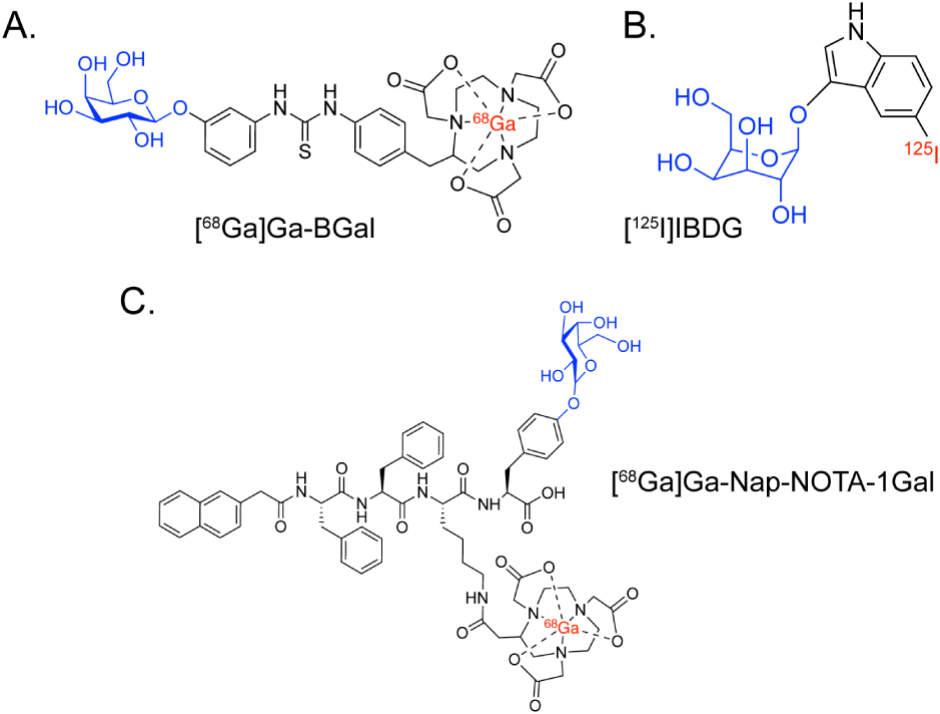
Structures of (A) [^68^Ga]­Ga-BGal, (B) [^125^I]­IBDG, and (C) [^68^Ga]­Ga-Nap-NOTA-1Gal.

#### Fibroblast Activation Protein

Fibroblast activation protein (FAP) is a dipeptidyl peptidase that has attracted significant attention in recent years due to its abundant expression by cancer-associated fibroblasts within solid tumors.^124–128^ Several small molecule- and antibody-drug conjugates have been developed to exploit the FAP-mediated release of toxins (Figure 15I). The nuclear medicine community has been particularly (and appropriately) enthralled with FAP as a pan-tumor target for both nuclear imaging and therapy, with tracers such as [^68^Ga]­Ga-FAPI-04 and [^68^Ga]­Ga-FAPI-46 in advanced clinical trials.^129–131^ However, there are presently no extant reports of nuclear imaging agents or radiotherapeutics that include FAP-cleavable linkers.

### Linkers Cleaved by Non-Enzymatic Mechanisms

As we have seen, enzymes can be effective tools for the cleavage of linkers in ADCs and radioconjugates. However, the heterogeneity of protein expression within disease presents an inherent—and unavoidable—limitation to this approach. In response, a wide variety of pharmaceuticals have been developed that are activated (i.e., cleaved) by nonenzymatic mechanisms. We will discuss these in the following pages.

#### Reducing Conditions (i.e., Glutathione)

The significant difference in the redox potential of the intracellular and extracellular environments as well as the elevation of glutathione levels in (rapidly dividing) cancer cells has spurred interest in the development of probes that release cargoes upon reduction. Along these lines, ADCs with disulfide linkages provide the most canonical example (Figure 21A). Mirvetuximab soravtansine (Elahere), for example, is an FDA-approved ADC for folate receptor-alpha-positive (FRα), platinum-resistant epithelial ovarian cancer that uses a disulfide linker to facilitate the cytosolic cleavage of DM4 in the cytosol of cancer cells, where glutathione concentrations are approximately 1000-fold higher than in plasma.^132,133^ Indeed, two FDA-approved ADCs—gemtuzumab ozogamicin (MyloTarg) and inotuzumab oxogamicin (Besponsa)—also include disulfide linkages that rely upon elevated glutathione concentrations within the cell to trigger the release of their toxins. Moving beyond disulfides, other groups have created novel approaches to the reduction-triggered release of cargoes. Tong et al., for example, developed a new class of reducible cleavable linkers based on the reduction-triggered 1,4-conjugation-addition reaction between a reduced azide and a cyclic α-alkylidene-β-diketone moiety (Figure 21B).^132^ The authors investigated the *in vitro* EGFR-targeting peptide-drug and antibody-drug conjugates bearing this linker motif, though no *in vivo* studies were described.

**Figure 21:**
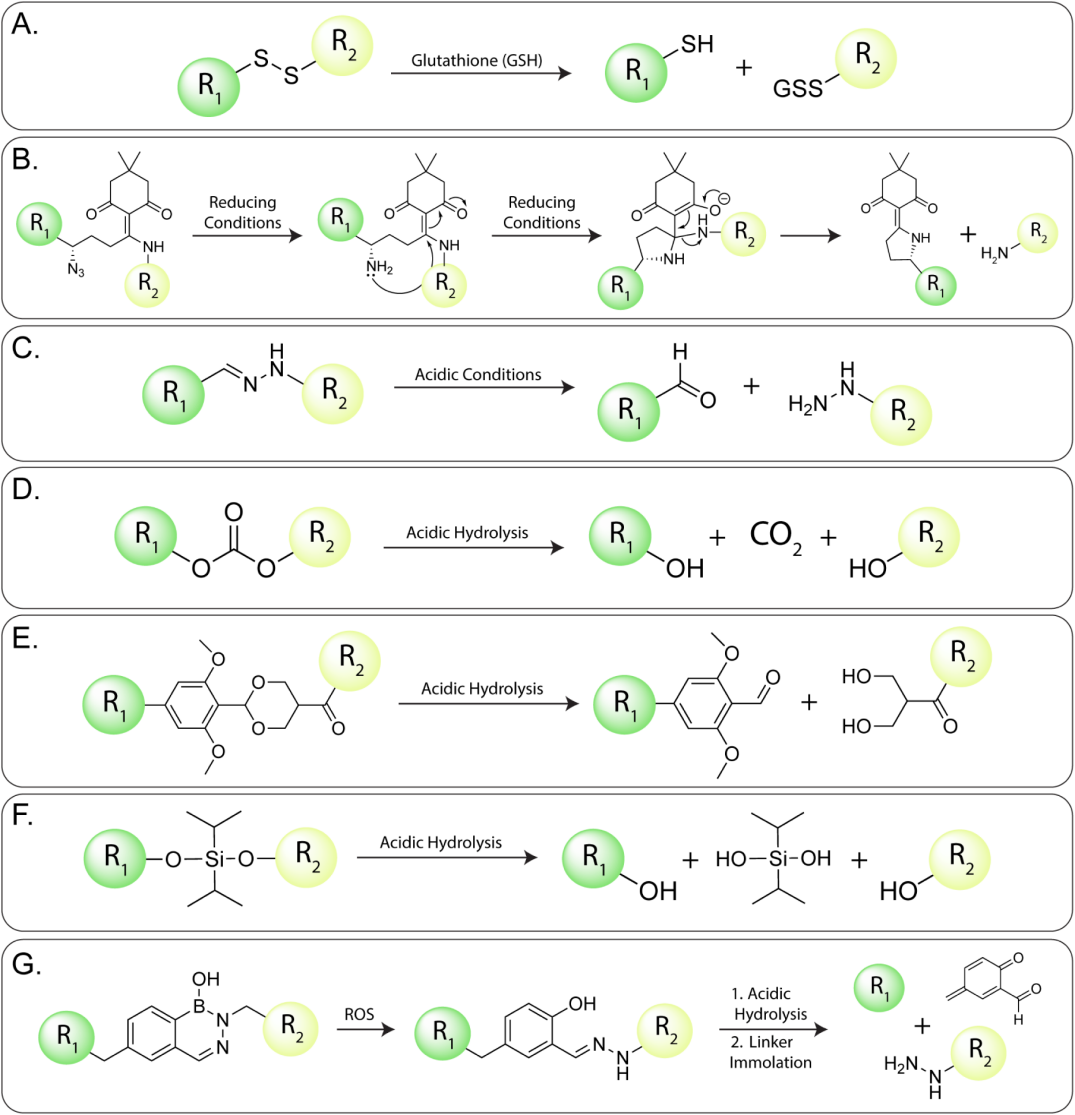
Schematics of the cleavage of dynamic linkers via endogenous (A) glutathione, (B) reducing conditions, (C–F) acidic conditions, and (G) reactive oxygen species. R_1_ and R_2_ represent payloads.

Given that the intracellular release of the radioactive cargo would not necessarily improve the performance of agents for nuclear imaging or therapy, there are few extant radiopharmaceuticals that employ disulfide linkers. Murce et al. recently created ^111^In- and ^177^Lu-labeled small-molecule drug conjugates in which a PSMA-targeting glutamate-urea-lysine vector is attached to DM1 via a disulfide linkage.^134^ While these probes exhibited “blockable” binding and selective cytotoxicity with PSMA-expressing cells, *in vivo* SPECT imaging studies revealed very low levels of tumoral uptake, suggesting that the attachment of the toxin dramatically alters the pharmacokinetic profile of the small molecule. Of course, the disulfide in this case does not link the radionuclide to the vector, but the literature does include one example of this type of architecture. In 2013, Cornelissen et al. described the development of a γH2AX-targeted nuclear localization sequence-bearing ^111^In-labeled mAb that was attached to EGF via a cleavable (i.e., disulfide) or static (i.e., PEG) linker.^135^ Interestingly, only the radioimmunoconjugate containing the cleavable linker was observed to associate with γH2AX foci within the nuclei of irradiated cells, a phenomenon that the investigators posited was responsible for the enhanced cytotoxicity of this compound compared to its noncleavable analogue in clonogenic assays.

#### Acidity

The acidic nature of lysosomes (pH 4.5–5.0) as well as the lowered pH of the tumor microenvironment (pH 6.0–7.0 compared to 7.4 for normal tissue) have fueled the use of acid-sensitive linkers in prodrugs and ADCs. Along these lines, the most common acid-cleavable linker moiety is the hydrazone, which decomposes to a hydrazine and a ketone or aldehyde in the presence of acid (Figure 21C).^4^ Hydrazones can be used in combination with other cleavable moieties—e.g., alongside disulfides in gemtuzumab ozogamicin and inotuzumab oxogamicin—or alone, as they are in the PD-L1-targeting, doxorubicin-bearing ADC developed by Sau et al.^136^ Carbonate linkages have also been used to facilitate the release of toxins at lowered pH, as in the FDA-approved ADC sacituzumab-govitecan (Trodelvy) (Figure 21D). And in recent years, ADCs have emerged bearing several other innovative acid-sensitive linkers, including cyclic acetals^137^ and silyl ethers^138^ (Figure 21E and F).

A variety of radiopharmaceuticals have been developed to target the low pH of the tumor microenvironment both directly [e.g., ^64^Cu- and ^18^F-labeled pH (low) insertion peptides] and indirectly (e.g., carbonic anhydrase IX-targeting [^89^Zr]­Zr-girentuximab). However, reports of radiopharmaceuticals bearing pH-responsive linkers are few and far between. In 2011, Wilbur et al. provided the most straightforward example via the creation of a library of probes in which acid-sensitive hydrazone linkers were used to combine a PSMA-targeting Fab with ^211^At-, ^125^I-, or ^131^I-labeled *closo*-decaborate (2^–^) moieties (Figure 22).^139^ Curiously, while the authors found that the hydrazone-bearing linkers accelerated the clearance of the radio*iodinated* prosthetic groups from the kidneys, they did not function similarly with the ^211^At-labeled *closo*-decaborates. More recently, Goos et al. created pH-responsive polymers in which linear poly­(*N*-(2-(hydroxypropyl)­methacrylamide)) were attached via pH-sensitive acyl hydrazone moieties to a variant of the hypoxia-targeted PET probe [^18^F]­FMISO.^140^ The investigators successfully demonstrated that the PET tracer was preferentially released in cells grown under acidic conditions *and* that enhanced uptake of the liberated [^18^F]­FMISO could be seen in hypoxic cells. However, no comparative *in vitro* experiments were performed with polymers containing noncleavable linkers, and no *in vivo* studies were reported. Finally, the Boros Laboratory recently reported the development of an innovative and elegant approach to the controlled release of metallodrugs based on metal-mediated autolytic amide bond cleavage.^141^ To date, the authors have employed this strategy only for the preparation of high-specific-activity radiopharmaceuticals, but the accelerated rate of the autolytic cleavage reaction under acidic conditions suggests that this chemistry could be leveraged to create acid-sensitive linkers.

**Figure 22:**
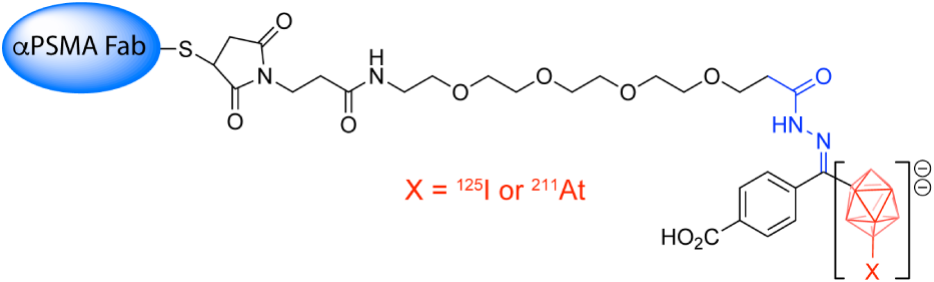
Schematic of a radioimmunoconjugate predicated on a PSMA-targeting Fab attached to an ^211^At- or ^125^I-labeled *closo*-decaborate­(2^–^) moiety via an acid-sensitive hydrazone linker (blue).

#### Reactive Oxygen and Nitrogen Species

Reactive oxygen species (ROS) and reactive nitrogen species (RNS) are, as their names suggest, highly reactive and unstable variants of molecular oxygen (e.g., H_2_O_2_, ^1^O_2_, O_2_^–^, and OH•) and nitrogen (e.g., NO, ONOO^–^, and NO_2_^–^) that are frequently products of aberrant metabolic processes and can damage sensitive biomolecules. ROS and RNS are well-established hallmarks of cancer and, as such, have attracted attention as triggers for drug conjugates and targets for imaging. With respect to the former, Antonio et al. recently developed an ADC in which the release of an SN38 toxin is modulated by a diazaborine-based linker than can be cleaved by ROS to unmask an acid-sensitive hydrazone (Figure 21G).^142^ With regard to the latter, several laboratories have created fluorogenic probes that “turn-on” upon selective reaction with ROS and/or RNS.

Given the critical role of ROS and RNS in cancer, it is not surprising that several PET and SPECT probes have been developed to enable their visualization. These agents, like the ADCs and fluorescent imaging probes discussed above, typically rely upon the reactivity of the ROS and RNS. Over the past decade, a team from the University of Pennsylvania has worked on the creation of cell-permeable dihydroethidium-based PET tracers that can be selectively oxidized by intracellular superoxide, thereby producing charged, aromatic species that become trapped in the cell.^143^ In one important example, the team demonstrated the specificity of one such ROS-reactive agent compared to a “preoxidized” variant in a murine model of doxorubicin-induced cardiac inflammation. Just last year, Wilde et al. reported the creation of [^18^F]­fluoroedaravone ([^18^F]­FEDV), a PET tracer that contains a 3-methyl-2-pyrazolin-5-one ring that—in the presence of intracellular ROS—undergoes a ring-opening reaction to produce a charged 2-oxo-3-(hydrazono)­butanoic acid species that becomes trapped in the cell.^144^ In this elegant work, the team used this probe to visualize ROS in several pathologies of the CNS. Despite this exciting work, there are no extant reports of PET or SPECT radioconjugates with ROS- or RNS-sensitive linkers, and the application of this chemistry in the context of therapeutic radionuclides is unlikely (given that the radionuclides themselves produce ROS and RNS).

### Linkers Cleaved by Exogenous Stimuli

As we have seen, linkers cleaved by endogenous enzymes and small molecules offer a potent approach to improving the safety and efficacy of radiopharmaceuticals. However, the reliance on endogenous triggers inevitably sacrifices precise control over *where* and *when* the linker is cleaved. To wit, a cathepsin-targeted linker can be cleaved anywhere in the body that it encounters the enzyme; in contrast, an RBBE-sensitive linker will only be cleaved in the kidneys, but this can occur whenever the radiopharmaceutical passes through the kidney tubules. In an effort to circumvent these limitations, the field has increasingly turned toward the use of linkers that can be cleaved by exogenously delivered stimuli. Broadly speaking, these linkers are designed to serve the same purposes as those discussed above, specifically accelerating the clearance of radioactivity from nontarget tissues and potentiating the efficacy of radiotherapeutics. However, exogenous stimuli offer several advantages over their endogenous counterparts, including (i) greater temporal control over cleavage (i.e., they can be administered at a precise moment after the injection of the radiopharmaceutical) and (ii) a lower likelihood of accidental cleavage by competing biomolecular triggers. Taken together, the exogenous triggers that have been explored to date fall into three categories—chemical, enzymatic, and electromagnetic—and each will be addressed in the following pages.

### Chemical Triggers

The rapid pharmacokinetic profiles and low immunogenicity of small molecules have made them particularly attractive triggers for the *in vivo* cleavage of linkers. Chemically induced scission strategies must generally satisfy two criteria: (i) the reaction must prompt the rapid release of the cargo and (ii) the two components of the reaction must be *bioorthogonal* (i.e., neither should react with any other components of the complex biological milieu). In light of these requirements, click chemistry transformations have garnered significant attention for the development of these approaches.^145,146^

Without question, the most popular click reaction exploited for the chemically triggered cleavage of linkers is the inverse electron-demand Diels–Alder (IEDDA) reaction between tetrazine (Tz) and *trans*-cyclooctene (TCO). Critically, the aromatization of the initial dihydrodipyridine product of the IEDDA ligation can trigger the release of a moiety attached to the TCO via a carbonate linkage (Figure 23A). This “click-to-release” strategy was originally developed by Rossin and Robillard with ADCs in mind, but it has been exploited in radiopharmaceutical chemistry several times since its discovery. In 2023, for example, Vlastar et al. reported the first in vivo demonstration of this technology.^147^ The team created a radioimmunoconjugate in which trastuzumab was linked to a [^89^Zr]­Zr-DFO moiety via a TCO-containing linker. PET imaging experiments in a murine model of HER2-expressing breast cancer revealed that the administration of a dipyridyl-Tz trigger 6 h after the administration of the ^89^Zr-labeled mAb effectively cleaved the radioimmunoconjugate’s linker, resulting in rapid accumulation of signal in the bladder as well as dramatically increased tumor-to-blood activity concentration ratios (Figure 24). A year later, Quintana et al. reported a very similar strategy but with “Scission-Enhanced Molecular Imaging (SEMI)” branding.^148^ Here, the investigators employed a TCO-bearing linker to bridge trastuzumab and a [^64^Cu]­Cu-DOTA moiety. The administration of a positively charged Tz trigger 4 h after the administration of the radioimmunoconjugate accelerated the clearance of radioactivity from the blood, but the increase in the tumor-to-blood activity concentration ratio compared to an analogous radioimmunoconjugate with a static linker was not statistically significant.

**Figure 23:**
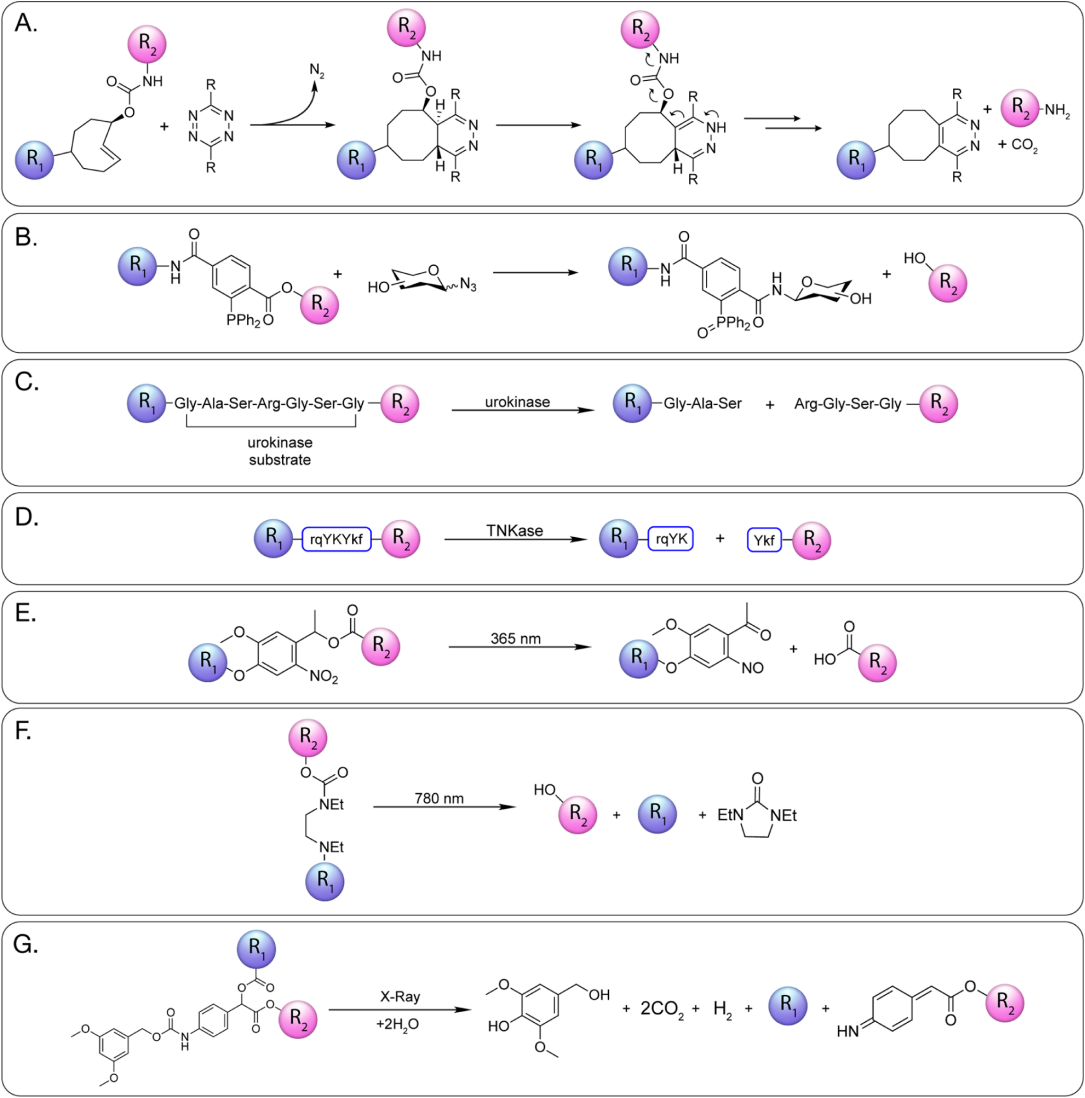
Schematics of linkers cleaved by (A) the inverse electron-demand Diels–Alder reaction; (B) the Staudinger ligation; (C) urokinase; (D) TNKase; (E) UVA radiation; (F) NIR radiation; (G) ionizing radiation. R_1_ and R_2_ represent payloads.

**Figure 24:**
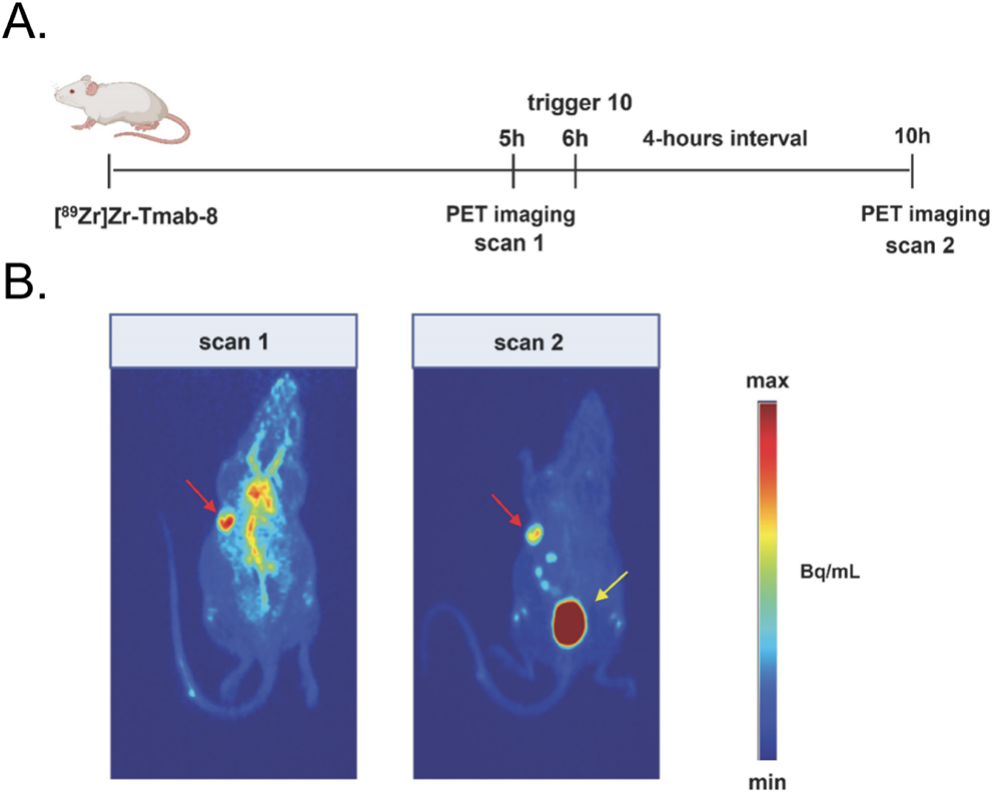
(A) Schematic of Vlastar et al.'s PET study using [^89^Zr]­Zr-trastuzumab-8 and mice bearing BT-474 xenografts. (B) Scan 1 (left) is collected prior to the administration of the trigger, whereas Scan 2 (right) is collected 4 h after the injection of the trigger. The red arrow indicates the tumor, while the yellow arrow indicates the bladder. Adapted and reprinted from ref^148^ under a Creative Commons License (CC BY 4.0).

The most wide-ranging and rigorous examination of this technology was reported just last year by Sarkar et al.^149^ This team created variants of rituximab in which three different radionuclides (i.e., iodine-125, zirconium-89, or gallium-68) were attached to the mAb via TCO-containing linkers and then explored these radioimmunoconjugates *in vivo* with five different Tz-based triggers. PET imaging and biodistribution experiments in a murine model of B-cell lymphoma suggest that this strategy may not be suitable for use with short-lived isotopes (or, at the very least, will require substantial optimization), as the administration of a Tz trigger 2 h after the injection of the ^68^Ga-labeled mAb actually *reduced* uptake in the tumor compared to the injection of vehicle alone. However, the *in vivo* results with the ^89^Zr-labeled radioimmunoconjugate were substantially better: the injection of a Tz trigger 24 h after the administration of [^89^Zr]­Zr-rituximab did not affect tumoral activity concentrations but did reduce the retention of radioactivity in several healthy tissues, including blood.

Shifting gears slightly, in 2024, Soni et al. used another bioorthogonal click reaction—the Staudinger ligation—to facilitate the *in vivo* cleavage of linkers (Figure 23B).^150^ Here, the investigators synthesized a radioimmunoconjugate of trastuzumab, in which a phosphine-containing linker appended an ^131^I-labeled prosthetic group to the mAb. A small library of *N*-glycosyl azides was tested as chemical triggers, and it was determined that PEGylated azides produced the most rapid cleavage of the payload *in vitro* (though this trend was *not* observed *in vivo*). Biodistribution experiments in mice bearing HER2-positive NIH3T6.7 xenografts found that the administration of azide-bearing variants of maltose and glucose 1, 3, and 5 h after the injection of [^111^I]­I-trastuzumab significantly reduced residual radioactivity levels in the blood without substantially affecting uptake in other healthy tissues (Figure 25). While these results are promising, the need for three injections of the chemical trigger does not necessarily bode well for clinical translation.

**Figure 25:**
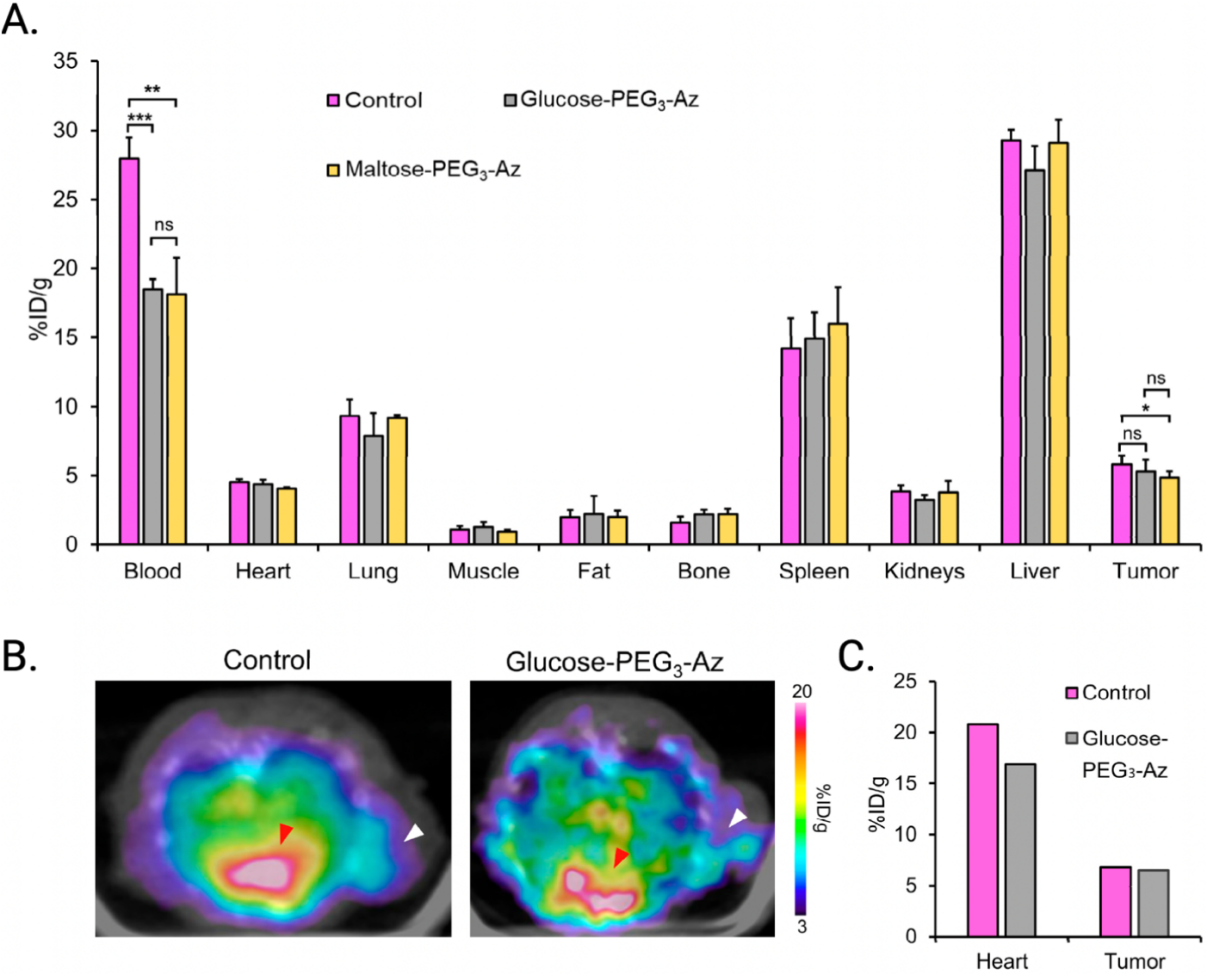
Biodistribution (A), SPECT (B), and image-derived region-of-interest analysis (C) data collected 7 h after the administration of [^131^I]­I-DTPA-trastuzumab to mice bearing NIH3T6.7 tumors, with doses of azide-modified saccharides injected 1, 3, and 5 h after that of the radioimmunoconjugate. (C) Red arrows indicate the heart, while white arrows indicate the tumor. Adapted and reprinted from ref^150^ under a Creative Commons License (CC BY 4.0).

### Enzymatic Triggers

As we have seen, enzymes have frequently been employed as *endo*genous agents for the cleavage of linkers. However, they can also be administered as *exo*genous triggers, a strategy that confers far greater temporal control over the cleavage reaction. In 2019, for example, Ren et al. developed a ^64^Cu-labeled variant of trastuzumab bearing a peptidic linker that could be cleaved by urokinase, an FDA-approved thrombolytic drug that dissolves blood clots by activating plasminogen (Figure 23C).^151^ In an effort to accelerate the clearance of circulating radionuclide from the blood, the intravenous administration of the radioimmunoconjugate was followed 24.5, 25.5, and 26.5 h later by three injections of urokinase. Subsequent PET imaging experiments in murine models of HER2-positive human epidermoid carcinoma revealed that the administration of urokinase dramatically improved the radioimmunoconjugate’s tumor-to-blood activity concentration ratios, yielding contrast at 24 h comparable to that achieved at 72 h with traditional radioimmunoconjugates (Figure 26). Despite its clear efficacy, this strategy nonetheless has several translational challenges, most notably the need for several injections of the exogenous agent and the fact that it requires doses of urokinase well above those typically used in the clinic. The authors hypothesize that endogenous urokinase inhibitors such as PAI-1 may be responsible for the latter issue; they propose the coadministration of PAI-1 inhibitors, but this would only increase the complexity of the system.

**Figure 26:**
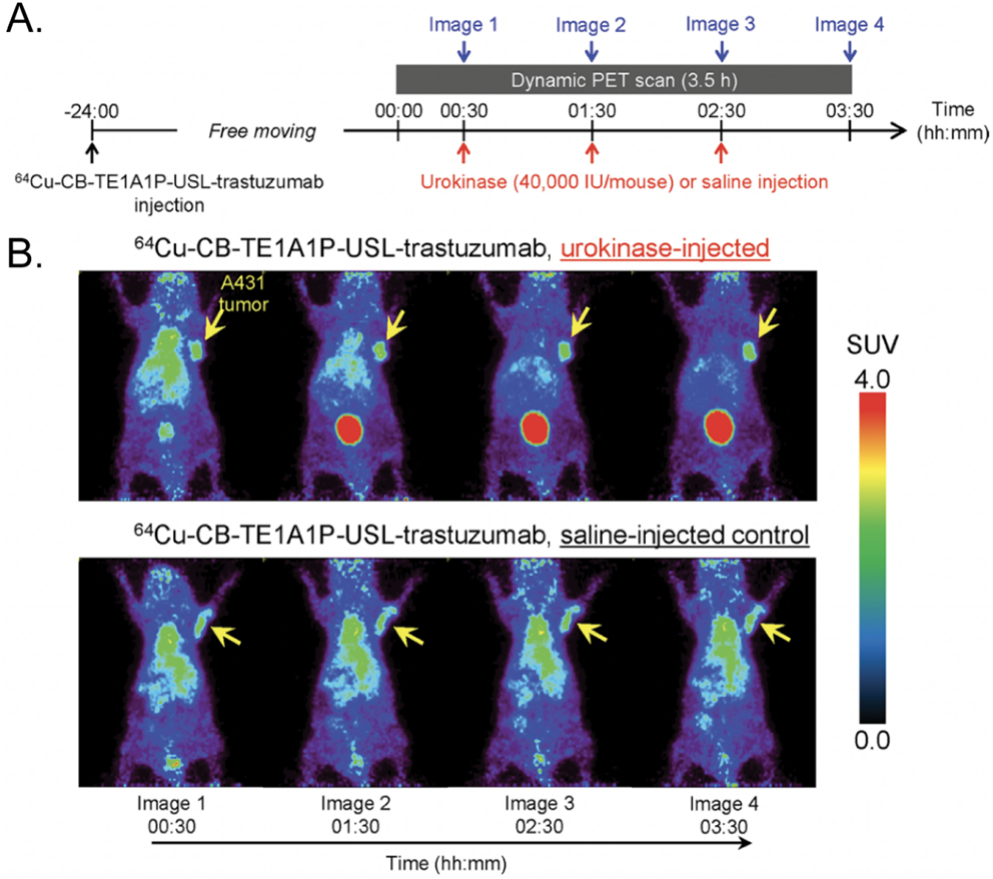
(A) Schematic of Ren et al.’s PET study in which mice bearing A431 xenografts were first administered [^64^Cu]­Cu-CB-TE1A1P-USL-trastuzumab followed by three injections of urokinase. (B) Coronal PET scans illustrating the differences between the cohort that received injections of urokinase (top) and the cohort that received injections of saline (bottom). Adapted and reprinted with permission from ref^151^.

A similar though more modest attempt at this sort of approach was made over a decade earlier by Kumaresan et al., who developed a peptidic linker that could be cleaved by TNKase, an FDA-approved plasminogen activator similar to urokinase (Figure 23D).^152^ The team constructed a radioimmunoconjugate in which an integral membrane glycoprotein-targeting mAb, ChL6, was linked to an [^111^In]­In-DOTA moiety via this cleavable linker and found that this probe was immunoreactive, stable in serum, and—critically—could be cleaved efficiently by clinical doses of TNKase *in vitro*. Unfortunately, however, *in vivo* validation experiments were not included in this proof-of-concept and were not subsequently reported.

Ultimately, while enzymes, especially those used as FDA-approved drugs, have some potential as exogenously administered triggers, the drawbacks seem to outnumber the benefits, especially when compared with small molecules.

### Other Triggers

To date, only two types of exogenous triggers—small molecules and enzymes—have been used to cleave linkers within radiopharmaceuticals. However, as we learned in our discussion of endogenous agents, nuclear medicine can learn a great deal from the ADC community when it comes to cleavable linkers. Thus, we will briefly explore three more types of exogenous triggers that have been leveraged for the cleavage of linkers in ADCs but have yet to be harnessed for nuclear imaging or therapy: ultraviolet light, near-infrared light, and X-rays.

#### Ultraviolet (UV) Light

In 2017, Wong et al. used a thioacetal *ortho*-nitrobenzaldehyde (TNB) linker to attach doxorubicin to a folate receptor-targeting nanoparticle.^153^ During *in vitro* experiments with KB carcinoma cells, exposure to long-wavelength ultraviolet A (UVA) prompted the rapid release of doxorubicin and a dramatic increase in cytotoxicity. Subsequent experiments pairing this UV-activated nanoparticle with a nanocrystal containing protoporphyrin IX (which can release UV and visible light when irradiated with NIR light) were likewise promising. However, no compelling *in vivo* results were presented with either system. More recently, Li et al. created an ADC in which a UV-responsive *o*-nitro-benzyl-containing linker attached an MMAE toxin to the HER2-targeting mAb Mil40 (Figure 23E).^154^ While *in vitro* assays with BT474-HerDR and N87-HerDR cells determined that the cytotoxicity of the ADC increased upon UV-irradiation, subsequent *in vivo* experiments revealed substantial uptake in the liver, suggesting that hepatotoxicity may be a problem. Ultimately, while these reports hold some promise, the applicability of this strategy to radiopharmaceutical chemistry is suspect given the poor tissue penetration of UV light.

#### Near-Infrared (NIR) Light

A decade ago, Nani et al. leveraged a linker containing a heptamethine cyanine fluorophore for the NIR-mediated release of combretastatin A4 (CA4) from an EGFR-targeted ADC.^155^ During *in vitro* assays with MDA-MB-468 cells, irradiation with NIR light prompted the efficient release of the drug and correlated with an increase in the cytotoxicity of the ADC. The NIR-triggered release of the drug was also confirmed in subsequent *in vivo* assays, though ultimately the team concluded that the toxin itself (i.e., CA4) was insufficient for their applications. As a result, the same team returned five years later with a similar NIR-activated ADC bearing a more potent payload: duocarmycin. Critically, cohorts of tumor-bearing mice treated with the ADC *and* irradiated with NIR light exhibited greater tumor control and longer median survival times than cohorts administered the ADC but *not* irradiated. Most recently, Fukushima et al. developed an NIR-activated, CD25-targeting ADC bearing a duocarmycin toxin to target regulatory T cells (Figure 23F).^156^ In murine models of urothelial carcinoma, this team found that their ADC (in combination with PD-1 blockade) resulted in NIR-light-dependent tumor growth suppression, increased survival, and higher tumoral infiltration of cytotoxic T cells. At the core, the greater tissue penetration of NIR light makes these strategies more enticing than those predicated on UV light from a radiopharmaceutical perspective; however, the extant data remain relatively sparse and should thus be viewed with caution.

#### X-rays

In 2022, Quintana et al. explored ionizing radiation as a trigger for the *in vivo* cleavage of 3,5-dimethylbenzyl alcohol (DMBA) linkers within an EGFR-targeting ADC and an albumin-drug conjugate carrying either doxorubicin or MMAE (Figure 23G).^157^ In preliminary studies, about 50% of the toxin was released upon irradiation of the conjugates with clinically relevant doses of X-rays (i.e., 1–16 Gy). Unfortunately, however, no *in vivo* studies were presented, rendering the assessment of this technology’s clinical potential difficult. Of course, the translation of this technology to radiopharmaceuticals would be wrought with issues, given that many radionuclides emit X-rays themselves. However, we chose to include the strategy here simply for the sake of its novelty.

## Conclusion

It is our hope that in the preceding pages, we have conveyed the remarkable breadth of linker chemistries that have been used to improve the pharmacokinetic profiles of nuclear imaging agents and radiotherapeutics. As we wrap up our discussion of this fertile area of research, it is our prerogative as authors to highlight some particularly exciting technologies. Among the static linkers, while we understand that the exigencies of time, familiarity, and commercial availability often drive members of our field to PEGylated linkers, we are truly excited by the innovative work being done on zwitterionic linkers, and we look forward to these playing a larger role in the field—and moving to the clinic—in the years to come. Among albumin binders, IPBA is clearly the most effective and versatile choice, as it is chemically simple (unlike Evans Blue), eschews genetic engineering (unlike albumin-binding domains), and does not dramatically increase the hydrophobicity of probes (unlike long-chain fatty acids). Critically, this is borne out by the handful of studies in which IPBA-containing linkers have been compared to linkers bearing other albumin binders.^61,158^ And finally, among the dynamic linkers, the “click-to-release” options are especially promising, both because of the spatial and temporal control they offer over cleavage and due to the wealth of data available on IEDDA-based approaches to *in vivo* pretargeting.

Shifting our gaze to the future, there are several trends that we hope will take shape in the years to come. First, we feel compelled to stress the need for *comparative* studies—and especially *comparative in vivo* work—during the preclinical evaluation of new linkers. As we assembled this paper, we were surprised (and disappointed) by the number of publications that do not include comparisons to probes that lack the linker under examination, as these data are absolutely essential to accurately assessing the new linker technology. Of course, the onus for this rigor ultimately falls with investigators, but reviewers can help the field by making these experiments a requirement in reviewing manuscripts. Second, we hope that the field begins to make greater use of new approach methodologies (e.g., organs-on-a-chip, *in ovo* models, and microfluidic systems) during the evaluation of new linkers, as the time and expense of *in vivo* validation in murine models can be significant obstacles to the optimization of novel linkers. Finally, and most importantly, we hope more clinical studies with innovative linkers will be performed, as only data from patients can truly cement the medical value of these technologies. In the end, it is our sincere hope that this review serves not only to summarize the incredible amount of impactful and innovative research that has already been done by members of this field but also to inspire our community to continue and expand upon this valuable work and ultimately prove its clinical value.
